# The TetR-type regulator AtsR is involved in multidrug response in *Corynebacterium glutamicum*

**DOI:** 10.1186/s12934-022-01850-0

**Published:** 2022-06-21

**Authors:** Tao Su, Chengchuan Che, Jiyu Han, Yuying Zhao, Zihan Zhang, Guangdi An, Meiru Si, Can Chen

**Affiliations:** 1 College of Life Sciences, Qufu Normal University, Qufu, Shandong 273165 China; 2 Key Laboratory of Plant Genetics and Molecular Breeding, College of Life Science and Agronomy, Zhoukou Normal University, Zhoukou, Henan 466001 China

**Keywords:** *Corynebacterium glutamicum*, AtsR, Multidrug resistance, TetR-type regulator

## Abstract

**Background:**

The TetR (tetracycline repressor) family is one of the major transcription factor families that regulate expression of genes involved in bacterial antimicrobial resistance systems. NCgl0886 protein, designated as AtsR, is a member of the TetR family identified in *Corynebacterium glutamicum*, which is conserved in several species of the genera *Corynebacterium*, also including the well-known pathogen *C. diphtheriae.* AtsR is located at no far upstream of the identically oriented *ncgl0884* gene, encoding a putative multidrug efflux pump protein, and in the same operon with *ncgl0887*, encoding a resistance, nodulation and cell division (RND) superfamily drug exporter. However, the role of AtsR is not clearly understood.

**Results:**

Here we showed that dimeric AtsR directly repressed the expression of the *ncgl0887*-*atsR* operon, as well as indirectly controlled the *ncgl0884* transcription. Antibiotics and toxic compounds induced the expression of *ncgl0887*-*atsR* operon. A perfect palindromic motif (5΄-TGCAA-N_2_-TTGCA-3΄; 12 bp) was identified in the upstream region of *ncgl0887*-*atsR* operon. Electrophoretic mobility shift assays (EMSAs) demonstrated specific binding of AtsR to this motif, and hydrogen peroxide (H_2_O_2_) blocked binding. H_2_O_2_ oxidized cysteine residues to form Cys123-Cys187 intermolecular disulfide bonds between two subunits in AtsR dimer, which altered its DNA-binding characteristics and caused its dissociation, thereby leading to derepression of the drug efflux protein. Deletion of *ncgl0884* and *ncgl0887* increased the susceptibilities of *C. glutamicum* for several toxic compounds, but overexpression of *atsR* decreased the drug tolerance of *C. glutamicum*.

**Conclusions:**

Our study revealed that AtsR was a redox regulator that sensed oxidative stress via thiol modification. The results obtained here will contribute to our understanding of the drug response mechanism not only in *C. glutamicum* but also in the related bacteria *C. diphtheriae.*

**Supplementary Information:**

The online version contains supplementary material available at 10.1186/s12934-022-01850-0.

## Background

Drug-resistant microorganisms are a major worldwide health issue, as a number of important human pathogens have now developed various mechanisms that make them largely resistant to all currently available treatment regimens. One of the important resistance mechanisms, becoming increasingly important, was the use of membrane-bound drug efflux pumps that were involved in the extrusion of toxic antimicrobial compounds [1]. There are two types of efflux systems: protein systems that transport a specific toxic compound or class of toxins from cell, which export numerous structurally dissimilar compounds [2]; the latter type of transporter is involved in the export of a wide range of antimicrobial compounds, which can eliminate the effects of many kinds of drugs (multidrug resistance (MDR)) [3]. A large number of MDR transport proteins have been found in bacteria. Moreover, the expression of the majority of the bacterial drug transporter genes is known to be subject to be inducible and be controlled by transcriptional regulatory proteins [3]. The antimicrobial pumps which are known to be subject to regulatory controls typically belong to either the major facilitator superfamily (MFS) or resistance, nodulation and cell division (RND) superfamily [1]. In general, the confirmed regulators of bacterial drug transporter genes belong to one of four regulatory protein families, the AraC (arabinose C regulator), MarR (multiple antibiotic resistance regulator), MerR (mercury regulator), and TetR (tetracycline repressor) families [1]. Among these regulatory proteins, the TetR family is one of the most prevalent families of transcriptional regulators in bacteria. The TetR family of transcriptional factors are known to regulate expression of genes involved in bacterial efflux systems against antimicrobial compounds or drugs [4]. Protein that belongs to this family is known to act as transcriptional repressors, and they bind to their regulatory sequence and repress expression of target genes that are related to drug detoxification or export [4]. When the cellular level of toxic compounds increases, the repressor alters its DNA-binding ability and dissociates from the regulatory sequence, resulting in activation of antibiotic resistance of the bacterium.

*Corynebacterium glutamicum* is a nonpathogenic gram-positive bacterium used as an important industrial strain for amino acid, nucleic acid, organic acid, alcohol, and biopolymer production and is a key model organism for studying pathogen evolution, such as *Corynebacterium diphtheriae* and *Mycobacterium tuberculosis*, which are phylogenetically related to *C. glutamicum* [5]. Sequencing of entire *C. glutamicum* genome has been found to encode a large number of putative MDR transporter homologs. The majority of transporter proteins belong to the two largest transporter families, MFS and ATP-binding cassette (ABC). Moreover, analysis of *C. glutamicum* (3.3 Mbp) genomes using the GTOP protein structure prediction data base (available on the World Wide Web at spock.genes.nig.ac.jp/genome/gtop.html) indicated that these genomes encode 16 proteins that possess the TetR (tetracycline repressor)-type DNA-binding domain, but many of their functions remain to be experimentally investigated [6]. Biochemical characterization and the mechanism of regulatory action of TetR-type regulators in *C. glutamicum* have focused on l-methionine synthesis repressor McbR [7], resorcinol regulator RolR [8], aconitase repressor AcnR [9], *C. glutamicum* multidrug-responsive transcriptional repressor CgmR[3], phenylacetic acid regulator PaaR [10], biotin biosynthesis and transport regulator BioQ [11], oxidative stress response regulator OsrR [12], ammonium transporters regulator AmtR [13], and *C. glutamicum* stress-sensing regulator CssR [14] so far. Functional analyses of such novel TetR family proteins found by genomic analysis promoted the elucidation of drug resistance mechanisms in the bacteria and contribute to reduction of the threat of MDR. In the genome of *C. glutamicum* ATCC 13,032, a single putative TetR-family transcriptional regulator NCgl0886, named AtsR (antibiotic- and toxic compound-sensing regulator) due to the results described in this study, is organized in an operon with a putative drug exporter of the resistance, nodulation and cell division (RND) superfamily (NCgl0887). No far downstream from *atsR* is multidrug efflux pump gene (*ncgl0884*), orientated in the same direction from *atsR*. Moreover, like other TetR-type regulators identified to date, NCgl0886 possesses winged helix-turn-helix (wHTH) DNA-binding motif, which are embedded in larger DNA-binding domains. Most well-described TetR-type proteins are transcriptional repressors and their genes are ubiquitously found in the adjacency, or is part of the regulated gene cluster [1]. This genomic ensemble emphasizes the role of AtsR as a transcriptional repressor of a putative drug efflux pump in *C. glutamicum*. In this study, we reported the finding of a novel TetR transcription factor AtsR in *C. glutamicum*. AtsR was found to possess the wHTH DNA binding motif and to exert a regulatory role on the *ncgl0887*-*atsR* operon and *ncgl0884* gene. These results should help light on the understanding of the multifaceted adaptive response in *C. glutamicum*.

## Results and discussion

### The TetR-type regulatorAtsR is conserved in *Corynebacteria*

The 582-bp *C. glutamicum ncgl0886* encoded a protein of 193 amino acids (mass, 21,459 Da), which has been predicted as a putative transcriptional regulator of the TetR family [15]. The role of this protein has not been studied hitherto. After further study, we renamed NCgl0886 as AtsR (antibiotic- and toxic compound-sensing regulator) based on the observed phenotypes presented below. Pfam analysis displayed that the deduced AtsR protein possessed the TetR-type winged helix-turn-helix motif located near the N-terminal region (amino acid residues 18–57). Secondary structure of AtsR using Phyre^2^ showed a high similarity to the secondary structure of the TetR-type EthR from *Mycobacterium tuberculosis* consisting of nine α-helices, despite poor amino acid sequence similarity between these proteins (Additional file 1: Figure S1A). Moreover, their N-terminal DNA binding domains (DBDs) displayed a moderate level of sequence similarity (amino acid sequence identity of the domain was about 30%). In addition, sequence alignment of the AtsR homologs in several *Corynebacterial* species, such as *C. crudilactis*, *C. callunae*, and *C. deserti*, revealed that the primary sequences were highly conserved, particularly in multi-helical N-terminal part from position 18 to 57, where nearly 62% of the amino acids were identical (Additional file 1: Fig. S1B). Conspicuously, despite moderate amino acid sequence similarity between these proteins (about 35%), N-terminal region of AtsR also exhibited clear similarity to that of the prominent pathogen *C. diphtheriae* TetR (about 60% identity) (Additional file 1: Fig. S1C). This phenomenon was in good agreement with previous studies that members of the TetR family had a high conservation of sequences in the DBDs and a highly divergent C-terminal ligand-binding domains (LBDs). This confirmed that the AtsR protein also belonged to the same family of proteins.

### Roles of AtsR, NCgl0887 and NCgl0884 in stress response in *C. glutamicum*

In *C. glutamicum* genome, the first 14 bases of the open reading frame (ORF) of *atsR* overlapped with the last 14 bases of *ncgl0887* ORF (Additional file 1: Fig. S2A). Combined with RAR-Seq-based newtrans analysis, we speculated these two genes formed an operon structure. The conjecture was confirmed by reverse transcriptional PCR (Additional file 1: Fig. S3). NCgl0887 was annotated as a putative drug exporter of the RND superfamily. RND was one of the major families to which a number of bacterial drug efflux pumps belonged. *atsR* was located at no far upstream of *ncgl0884* gene encoding a putative multidrug efflux pump protein. Interestingly, the same gene organization was also found in *C. crudilactis*, *C. callunae*, *C. deserti*, and *C. efficiens* (Additional file 1: Figure S1D). This genomic ensemble emphasized a role of AtsR in multidrug resistance. To estimate the biological function of AtsR, we constructed *C. glutamicum atsR* deletion, the complementary strains and AtsR-overexpressing strains by gene disruption, complementation, or overexpression (Additional file 1: Fig. S2B), and then the MICs of toxic compounds for these strains were determined. A growth analysis on LB broth medium found that the WT(pXMJ19) strain, Δ*atsR*(pXMJ19) mutant and WT(pXMJ19-*atsR*) strain showed almost identical growth rates (Additional file 1: Fig. S4). When AtsR was overexpressed, the tolerance of *C. glutamicum* RES167 strain to several toxic compounds was obviously decreased (Table 1). Deletion of *atsR* did not affect the drug susceptibilities.

NCgl0887, the product of the *ncgl0887*, was composed of 791 amino acid residues. Amino acid sequence comparison showed that NCgl0887 was highly homologous to drug exporters of the RND superfamily (showing 91.8%, 97.1%, 91.2%, and 82.0% amino acid identities to exporters from *C. crudilactis*, *C. callunae*, *C. deserti*, and *C. efficiens*, respectively) (Additional file 1: Fig. S1D). NCgl0884 has sequence similarity with multidrug efflux pump proteins from other bacteria (Additional file 1: Figure S1D), such as *Pseudogulbenkiania* multidrug efflux pump transporter AcrB and *Zymomonas mobilis* MexB, which conferred cells resistant to multidrug resistance by a proton motive force-dependent mechanism [16, 17]. These results indicated that NCgl0887 and NCgl0884 may also possess strong potential to protect cells from drugs. As expected, although the Δ*ncgl0887*(pXMJ19) and Δ*ncgl0884*(pXMJ19) mutant did not affect bacterial growth under normal conditions (Additional file 1: Fig. S4), the Δ*ncgl0884*(pXMJ19) and Δ*ncgl0887*(pXMJ19) mutants were sensitive to several agents, as shown in Table 1. Moreover, the sensitive phenotype was almost completely reversed in the complementary strains Δ*ncgl0884*(pXMJ19-*ncgl0884*) and Δ*ncgl0887*(pXMJ19-*ncgl0887*) (Table 1), suggesting NCgl0884 and NCgl0887 protected cells from detrimental effects, which is in agreement with a previous study on NCgl0887 reported by Yang et al. [18] Yang et al. found that NCgl0887 protected cells from antibiotics by playing essential roles in the biogenesis of the low-permeability barrier of the outer membrane [18]. However, overexpression of *atsR* did not affect the drug susceptibility of Δ*ncgl0884* and Δ*ncgl0887* mutants.

### AtsR repressed the expression of *ncgl0884* gene and the *ncgl0887*-*atsR* operon

**Fig. 1:**
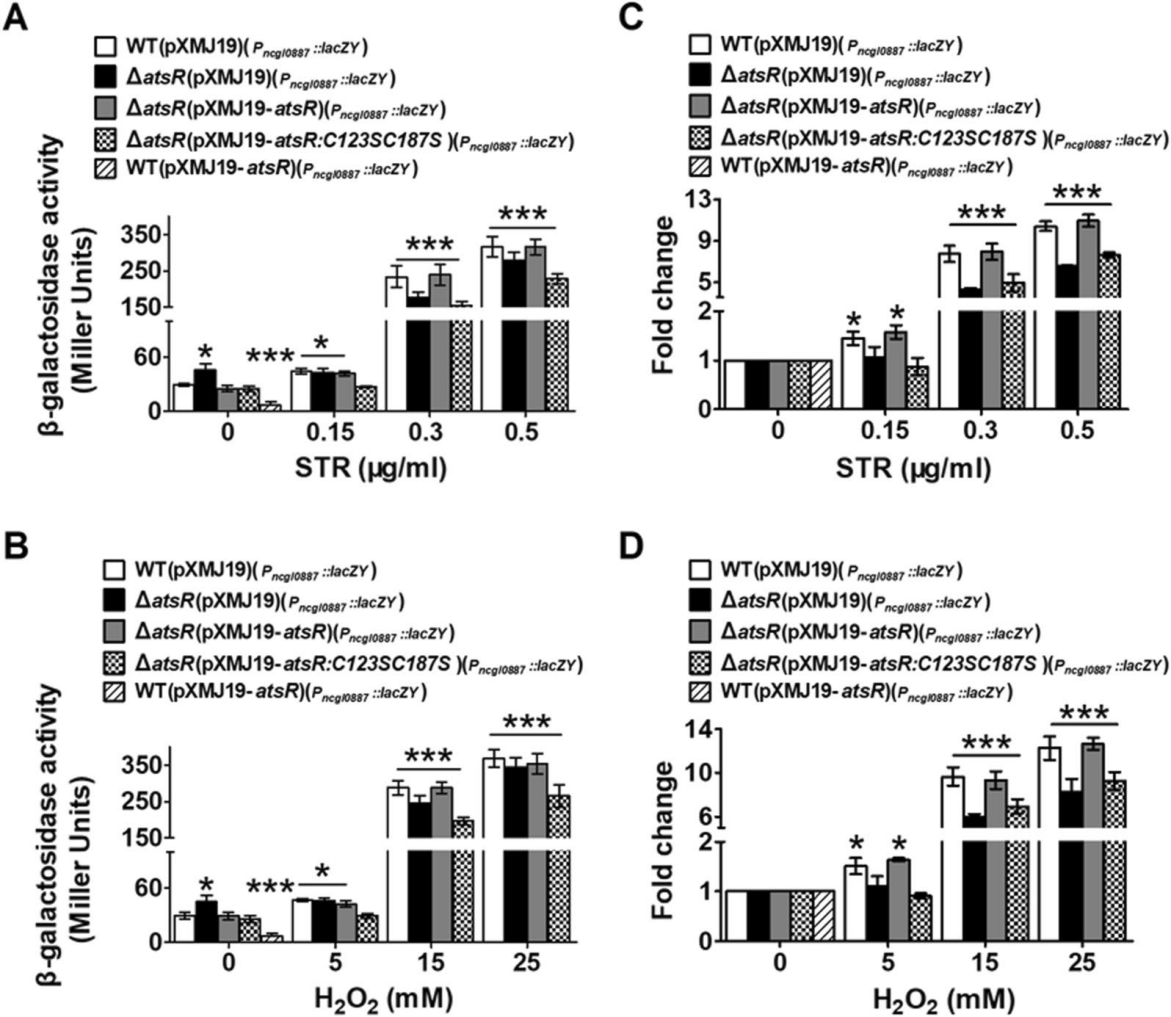
β-galactosidase activity driven by the promoter of the *ncgl0887*-*atsR* operon.** A, B** β-galactosidase activity analysis of the promoter was performed using the transcriptional *P*_*ncgl0887*_::*lacZY* chromosomal fusion reporter expressed in WT(pXMJ19), Δ*atsR*(pXMJ19), Δ*atsR*(pXMJ19-*atsR*), and Δ*atsR*(pXMJ19-*atsR:C123SC187S*)(the Δ*atsR* mutant expressed the pXMJ19-*atsR:C123SC187S*) strains exposed to streptomycin (STR) or hydrogen peroxide (H_2_O_2_) for 30 min. β-galactosidase activity analysis of the promoter in WT(pXMJ19-*atsR*) strains was performed in the absence of agents. **C, D** Fold change of the transcription level was calculated according to the data from Fig. 1A, B by the equation: the value obtained from the strains exposed to stress/the value obtained from the corresponding strains without stress

By PROM-Prediction of bacterial promoter and RAR-Seq-based newtrans analysis, a deduced promoter element [− 10 (TTAAGA) and − 35 (TTGTCC) regions] was found in upstream region of *ncgl0887* gene (Additional file 1: Fig. S2A). In several cases, TetR-type regulators were described to repress a small set of drug efflux genes, often located in the same operon or in the adjacency to the regulator gene on the chromosome [1]. In combined with the corresponding genomic loci (Additional file 1: Fig. S1D), we speculated that AtsR repressed the transcription of the *ncgl0887*-*atsR* operon and *ncgl0884* gene in *C. glutamicum.* To investigate this possibility, a single copy *P*_*ncgl0887*_::*lacZY* or *P*_*ncgl0884*_::*lacZY* fusion was introduced into the chromosomes of WT(pXMJ19), Δ*atsR*(pXMJ19), Δ*atsR*(pXMJ19-*atsR*), and WT(pXMJ19-*atsR*) strains and LacZY activity of the resulting strains was quantitatively measured. *P*_*ncgl0887*_ was used to represent the promoter of the *ncgl0887*-*atsR* operon. Figure 1A, B showed that the slightly increased levels of β-galactosidase activity (~ 1.5-fold) were observed in the Δ*atsR*(pXMJ19) mutant compared with those in the WT(pXMJ19) and Δ*atsR*(pXMJ19-*atsR*) strains; β-galactosidase activity was found to be lower in strain WT(pXMJ19-*atsR*) than in WT(pXMJ19) strain. This indicated that the promoter of the *ncgl0887*-*atsR* operon was negatively regulated by AtsR .

**Fig. 2:**
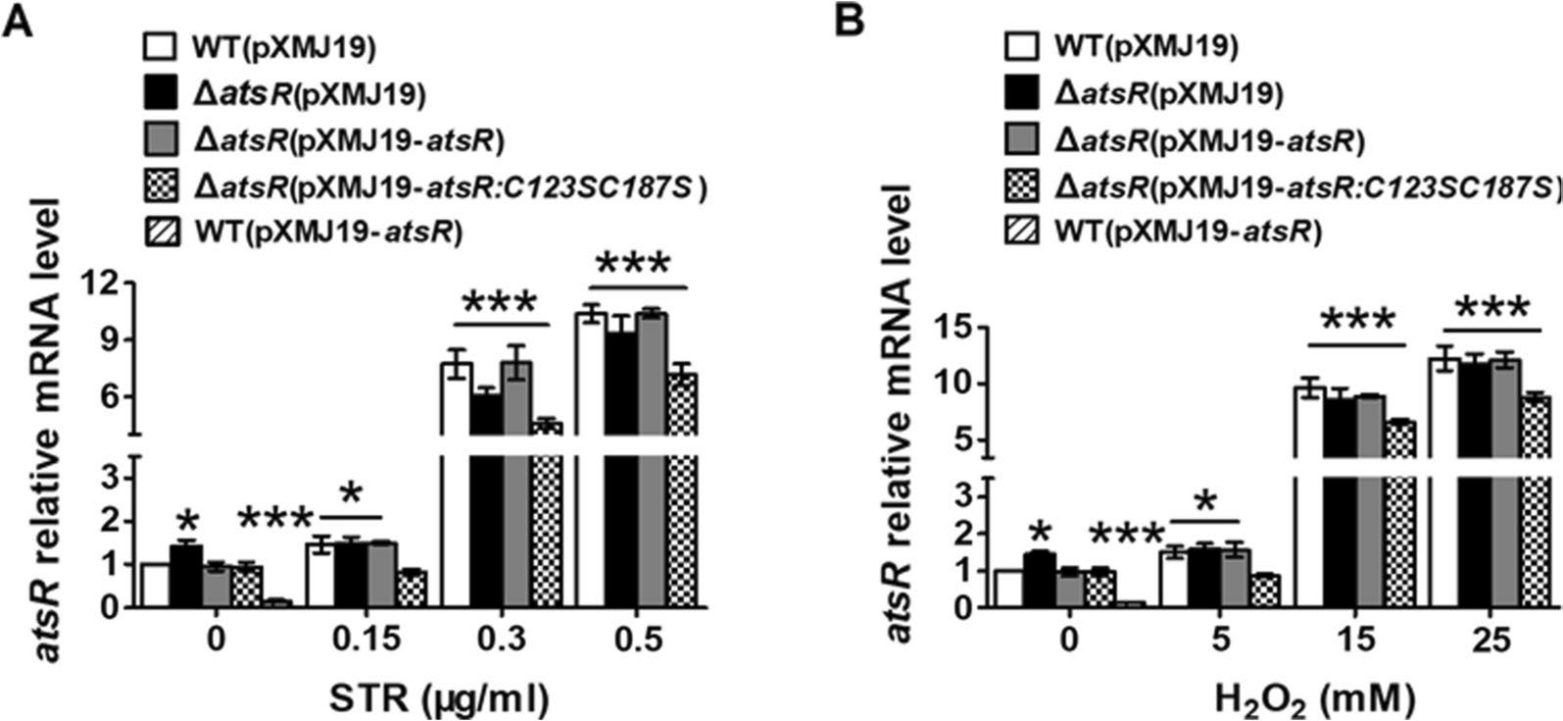
qRT-PCR analyses examining the transcription of *atsR* gene. qRT-PCR assay was performed to analyze the expression of *atsR* in WT(pXMJ19), Δ*atsR*(pXMJ19), Δ*atsR*(pXMJ19-*atsR*), and Δ*atsR*(pXMJ19-*atsR:C123SC187S*) strains exposed to STR (**A**) and H_2_O_2_ (**B**) for 30 min. qRT-PCR assay was performed in WT(pXMJ19-*atsR*) strains in the absence of agents. The mRNA levels were presented relative to the value obtained from WT(pXMJ19) strains without treatment. Relative transcript levels of WT(pXMJ19) strains without stress treatment were set at a value of 1.0. Data show the averages of three independent experiments, and error bars indicated the SDs from three independent experiments. ***P ≤ 0.001 and *P ≤ 0.05 for the indicated strains versus WT strain without stress treatment

**Fig. 3:**
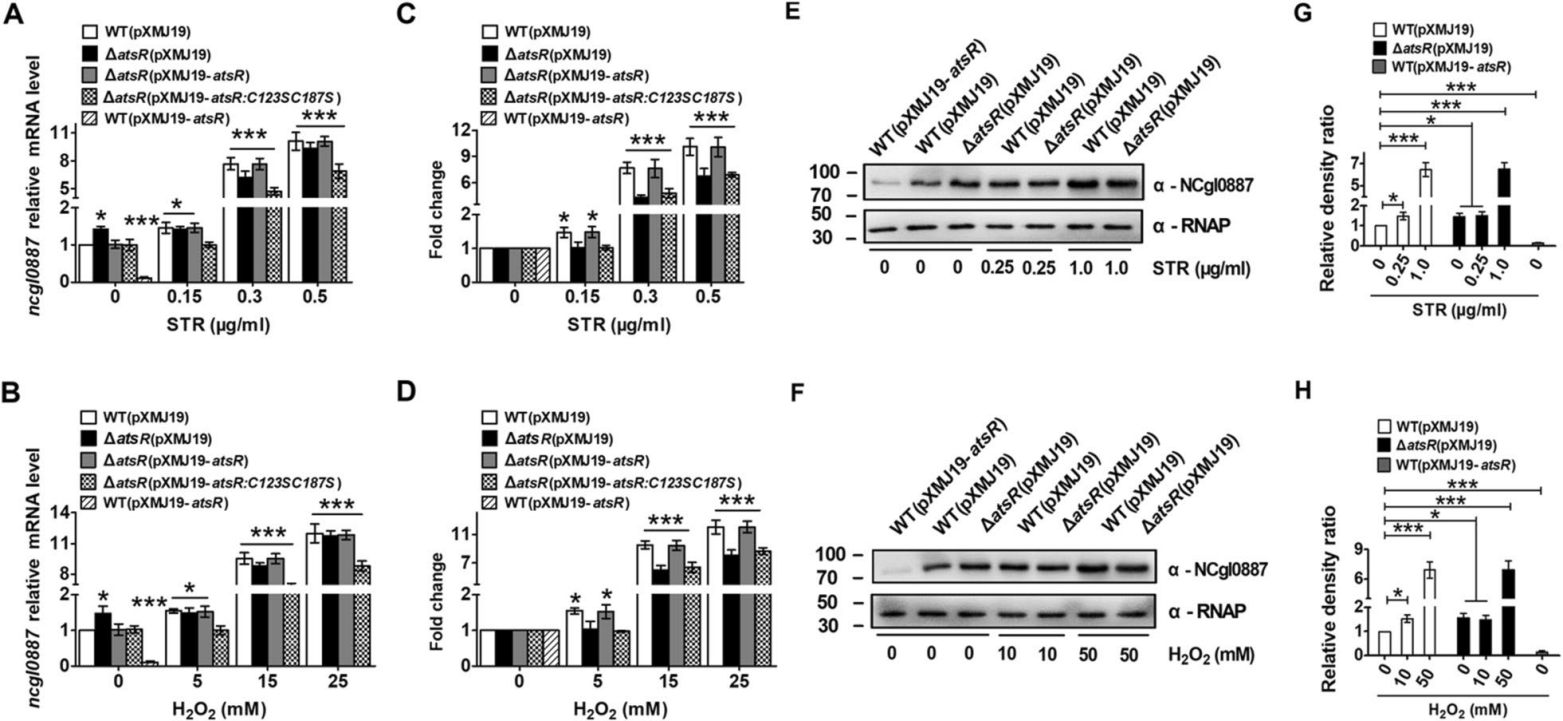
Negative regulation of *ncgl0887* expression by AtsR. A, B qRT-PCR assay was performed to analyze the expression of *ncgl0887* in WT(pXMJ19), Δ*atsR*(pXMJ19), Δ*atsR*(pXMJ19-*atsR*), and Δ*atsR*(pXMJ19-*atsR:C123SC187S*) strains exposed to STR and H_2_O_2_ for 30 min. qRT-PCR assay was performed in WT(pXMJ19-*atsR*) strains in the absence of agents. The mRNA levels were presented relative to the value obtained from WT(pXMJ19) strains without treatment. Relative transcript levels of WT(pXMJ19) strains without stress treatment were set at a value of 1.0. Data show the averages of three independent experiments, and error bars indicated the SDs from three independent experiments. ***P ≤ 0.001 and *P ≤ 0.05 for the indicated strains versus WT strain without stress treatment. **C, D** Fold change of the transcription level was calculated according to the data from** A**,** B** by the equation: the value obtained from the strains exposed to stress/the value obtained from the corresponding strains without stress. Data show the averages of three independent experiments, and error bars indicated the SDs from three independent experiments. ***P ≤ 0.001 and *P ≤ 0.05 for the indicated strains versus WT strain without stress treatment. **E**,** F** The protein levels of NCgl0887 in *C. glutamicum* corresponding strains in the presence or absence of STR and H_2_O_2_. Lysates from stationary phase bacteria exposed to STR and H_2_O_2_ for 2 h were resolved by SDS-PAGE, and NCgl0887 was detected by immunoblotting using specific anti-NCgl0887 antibody. For the pellet fraction, cytosolic RNA polymerase α (α-RNAP) was used as a loading control. Similar results were obtained in three independent experiments, and data shown were from one representative experiment done in triplicate. **G**, **H** Relative quantified data for protein levels by Image Lab. Quantified protein expression of western blots in** E**,** F**. Densities of proteins were all justified with α-RNAP. Relative density ratios of WT without stress were set at a value of 1.0. Data show the averages of three independent experiments, and error bars indicated the SDs from three independent experiments. ***P ≤ 0.001; *P ≤  0.05

qRT-PCR was also performed to further assess the effects of AtsR on the expression of *atsR* and *ncgl0887* genes. Notably, to study the expression of *atsR* in the Δ*atsR* mutant by qRT-PCR, an 86-bp *atsR* transcript (corresponding to nucleotides + 1 to + 86 relative to the translational start codon (GTG) of the *atsR* gene) was amplified from the remaining *atsR* ORF in the Δ*atsR* mutant with the primers QatsR-F and QatsR-R (Additional file 1: Fig. S5). As shown in Figs. 2A, B and 3A, B, the transcriptional levels of *atsR* and *ncgl0887* were slightly enhanced or significantly decreased in strains Δ*atsR*(pXMJ19) and WT(pXMJ19-*atsR*) compared with those in strain WT(pXMJ19) (~ 1.47-fold increase and 90% decrease, respectively). Further immunoblotting with anti-NCgl0887 antibody showed that overexpression of *atsR* obviously decreased the production level of the NCgl0887 protein in WT(pXMJ19) strain under normal condition, while the production level of NCgl0887 was slightly increased in cells lacking AtsR (Fig. 3E–H and Additional file 1: Fig. S6). Similarly, the enhanced β-galactosidase activity of the *ncgl0884* promoter and the increased mRNA levels of *ncgl0884* gene were observed in Δ*atsR*(pXMJ19) mutant compared with those in the WT(pXMJ19) strain (Additional file 1: Fig. S7), indicating that *ncgl0884* was negatively controlled by AtsR. Together, these results indicated that AtsR negatively controlled the expression of *ncgl0884*, *ncgl0887*, and its structural gene.

### The agent-induced expression of the *ncgl0887*-*atsR* operon

Previous biochemical and genetic characterizations indicated that the expression of multidrug efflux pump genes controlled by TetRs was induced by agents [4]. Thus, these studies led us to investigate whether AtsR participated in the induction of the *ncgl0887*-*atsR* operon by agents. For simplicity, we used streptomycin (STR) or hydrogen peroxide (H_2_O_2_) as inducers in the following experiments. As shown in Fig. 1A–D, in the absence of STR and H_2_O_2_, the Δ*atsR*(pXMJ19)(*P*_*ncgl0887*_::*lacZY*) strain had a mildly higher β-galactosidase activity (~ 1.45-fold) than the WT(pXMJ19)(*P*_*ncgl0887*_::*lacZY*) and Δ*atsR*(pXMJ19-*atsR*)(*P*_*ncgl0887*_::*lacZY*) strains. In strains WT(pXMJ19)(*P*_*ncgl0887*_::*lacZY*) and Δ*atsR*(pXMJ19-*atsR*)(*P*_*ncgl0887*_::*lacZY*) induced by high concentration of STR and H_2_O_2_ (0.5 µg/ml STR and 25 mM H_2_O_2_), β-galactosidase activity was approximately ten times higher than in the cells uninduced by STR and H_2_O_2_, while the only slightly enhanced activity was observed under low concentration inducers (0.15 µg/ml STR and 5 mM H_2_O_2_) (~ 1.5-fold). On the other hand, the level of β-galactosidase activity was found to be not increased in the Δ*atsR*(pXMJ19)(*P*_*ncgl0887*_::*lacZY*) strain under the low concentration of STR- and H_2_O_2_-induced conditions, which was maintained at the same level observed in the Δ*atsR*(pXMJ19)(*P*_*ncgl0887*_::*lacZY*) strain without STR and H_2_O_2_ treatment. However, 0.5 µg/ml STR and 25 mM H_2_O_2_ induced the significantly increased levels of β-galactosidase activity in the Δ*atsR*(pXMJ19)(*P*_*ncgl0887*_::*lacZY*) strain, showing a similar level to those in the WT(pXMJ19)(*P*_*ncgl0887*_::*lacZY*) and Δ*atsR*(pXMJ19-*atsR*)(*P*_*ncgl0887*_::*lacZY*) strains in response to high concentration of STR and H_2_O_2_ (Fig. 1A–D). The β-galactosidase activity observed under induction was consistent with the mRNA levels in the cells induced by STR and H_2_O_2_ (Figs. 2A, B, 3A, B). Further analysis at the protein level indicated that similar regulation was observed for the production of NCgl0887, in which low and high concentration of STR or H_2_O_2_ treatment mildly and strongly increased its level in WT(pXMJ19) strains, respectively, while low concentration of STR and H_2_O_2_ treatment did not affect the production level of NCgl0887 in Δ*atsR*(pXMJ19) (Fig. 3E–H). These results clearly demonstrated that the expression of the *ncgl0887*-*atsR* operon by AtsR in vivo was induced by antibiotics and toxic compounds and was likely also mediated by other stress-sensing regulators.

### AtsR directly bound the promoter of the *ncgl0887***-***atsR***operon**

**Fig. 4:**
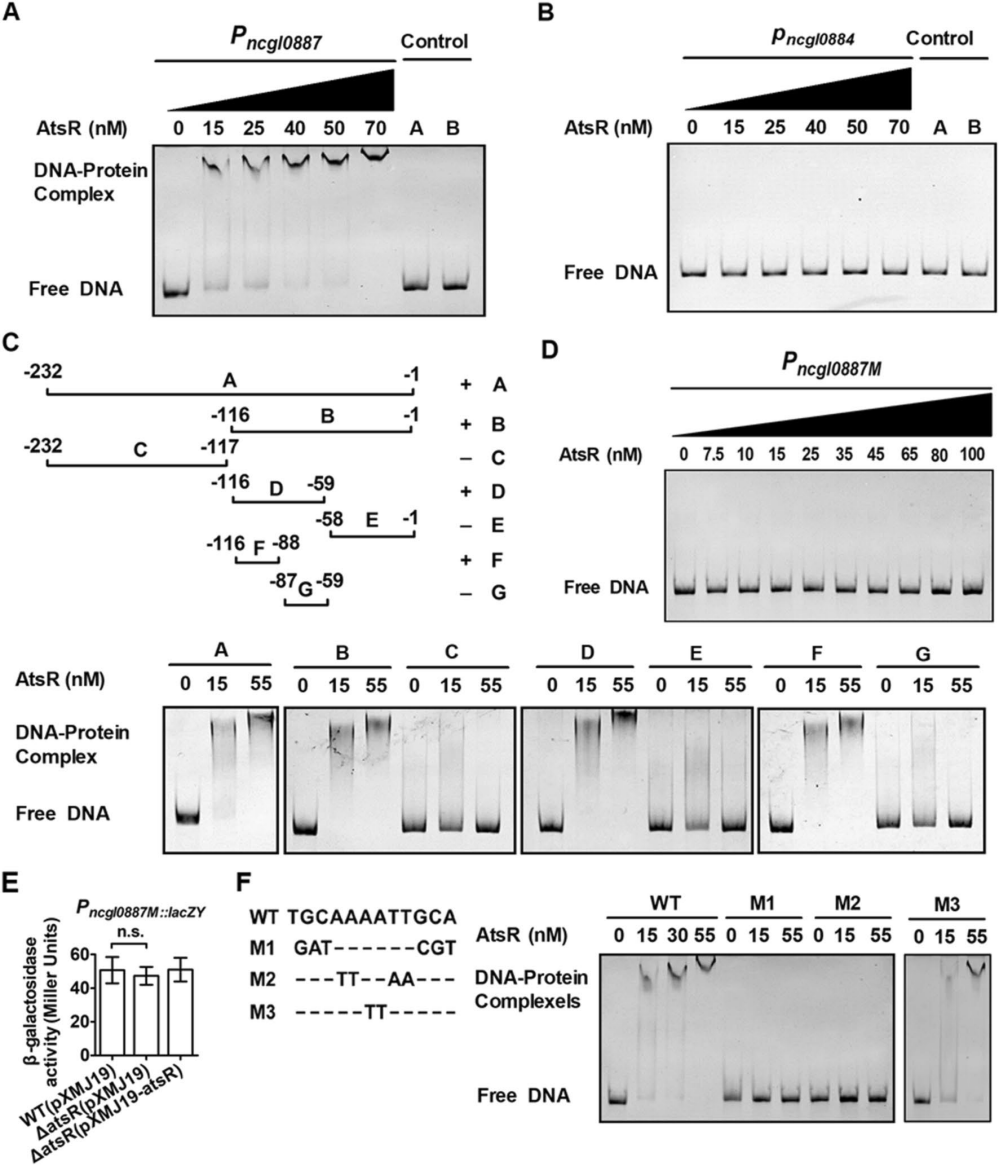
AtsR bound directly to the promoter region of the *ncgl0887-atsR* operon.** A**,** B** EMSA was performed to analyze the interaction between AtsR and the promoter DNA fragment of the *ncgl0887-atsR* operon (*P*_*ncgl0887*_) or the *ncgl0884* promoter DNA fragment (*P*_*ncgl0884*_). 232-bp and 205-bp DNA fragments amplified from the *ncgl0887* coding region using the primers control F1 and control R1 instead of the 232-bp *P*_*ncgl0887*_ (control A, lane 7) and the *ncgl0884* coding region using the primers control F2 and control R2 instead of the 205-bp *P*_*ncgl0884*_ (control A, lane 7), respectively, and an irrelevant protein BSA instead of AtsR (control B, lane 8) in the binding assays were used as negative controls to determine the binding specificity of AtsR. **C** Identification of the AtsR-binding sites within the *P*_*ncgl0887*_. Localization of the AtsR binding site using the *ncgl0887-atsR* operon promoter fragments (designated** A**–**G**) and purified AtsR in EMSAs. The *numbers* showed the position of the fragments relative to the translational start codon of the target gene. ‘+’and ‘−’ signs indicated whether the fragment was shifted by AtsR. **D** The interaction between AtsR and the promoter DNA fragment of the *ncgl0887-atsR* operon mutating the identified AtsR binding regions (*P*_*ncgl0887M*_). **E** Mutations in the identified AtsR binding site derepressed the expression of the *ncgl0887-atsR* operon. n.s., not significant. **F** The 12-bp proposed AtsR consensus binding site (bold). The mutations M1–M3 were introduced by PCR and were shown below the wild-type (WT) sequence. The corresponding DNA fragments were analyzed by EMSAs with AtsR

To investigate whether AtsR directly regulated the transcription of the *ncgl0887*-*atsR* operon and *ncgl00884* gene, we performed electrophoretic mobility shift assays (EMSAs) using purified AtsR and 232-bp *P*_*ncgl0887*_ or 205-bp *P*_*ncgl0884*_. As shown in Fig. 4A, incubation of *P*_*ncgl0887*_ with AtsR caused a clear delay in promoter DNA migration, and *P*_*ncgl0887*_ migrated in a manner dependent on the concentration of AtsR. This effect was specific because the combination of AtsR and DNA fragments amplified from the *ncgl0887* ORF did not delay migration (Fig. 4A, lane 7); incubation of BSA with *P*_*ncgl0887*_ did not lead to retarded mobility (Fig. 4A, lane 8). The apparent *K*_*D*_ value of *P*_*ncgl0887*_ was about 34 nM AtsR (Additional file 1: Fig. S8A), which was within the range found for other transcriptional regulators [19]. However, AtsR did not bind to the promoter region of the *ncgl0884* gene (Fig. 4B). To locate the binding site of AtsR in the promoter region of the *ncgl0887*-*atsR* operon, the 232-bp fragment A used for the initial binding studies was split into several smaller fragments, which were synthesized by PCR and analyzed in EMSAs for binding of AtsR. As shown in Fig. 4C, fragments B, D, and F were bound by AtsR. As fragments B and D contained fragment F. Thus, the most important part of the AtsR binding site was assumed to be located within these 29-bp fragment F, which extended from position − 116 to − 88 with respect to the translational start codon (GTG) of the *ncgl0887*-*atsR* operon. To confirm the conjecture, the 232-bp promoter DNA fragments of the *ncgl0887*-*atsR* operon containing the mutated 29-bp fragments (*P*_*ncgl0887M*_) were used for EMSA analysis (Additional file 1: Fig. S2A). As shown in Fig. 4D, *P*_*ncgl0887M*_ abolished the formation of DNA-protein complexes by the EMSA assay. Consistently, the mutations in 29-bp fragments led to the high β-galactosidase activities in the WT(pXMJ19)(*P*_*ncgl0887M*_::*lacZY*) and Δ*atsR*(pXMJ19-*atsR*)(*P*_*ncgl0887M*_::*lacZY*) strains, similar to that in the Δ*atsR*(pXMJ19)(*P*_*ncgl0887M*_::*lacZY*) mutant (Fig. 4E). In combined with the phenomenon that AtsR binding site was not found in the promoter region of *ncgl0884*, we speculated that AtsR regulated the *ncgl0887*-*atsR* operon and the *ngcl0884* gene directly and indirectly in *C. glutamicum*, respectively. These results further indicated that the corresponding sequence was required for AtsR binding.

### Identification of the AtsR binding motif

The binding sites of TetR-type regulators were featured by palindrome sequences [4]. Careful examination of 29-bp fragment F and three bases outside both sides of fragment F revealed a 5-bp perfect inverted repeat-TGCAAAATTGCA-which might be related to the AtsR operator. The relevance of this region was analyzed by mutational analysis. Figure 4F showed that fragments M1-M3 represented derivatives of fragment WT with mutations within the proposed binding motif. Fragments M1 and M2 completely inhibited the shift. In contrast, exchange of the two inner bases (fragment M3) did not prevent the shift. This result demonstrated that M1 and M2 corresponding nucleotides were required for the binding of AtsR. Thus, we proposed DNA sequence motif of AtsR was 5′-TGCAAAATTGCA-3′.

**Fig. 5:**
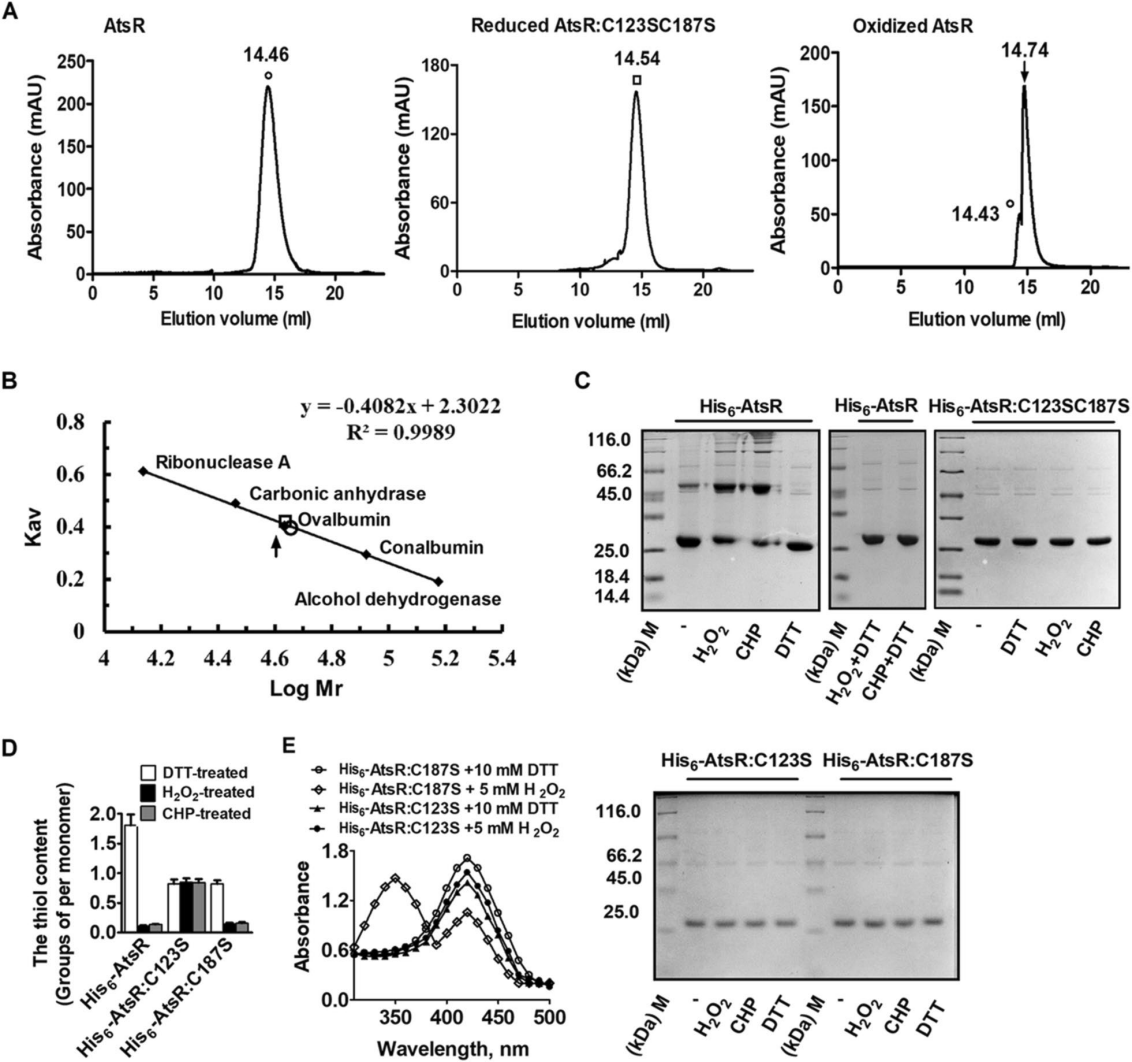
Determination of the native molecular mass by gel filtration.** A** Elution of native AtsR, reduced AtsR:C123SC187S, and oxidized AtsR from size exclusion column. Native AtsR and reduced AtsR:C123SC187SAtsR (20 µM AtsR was incubated with 50 mM DTT) were indicated by clear circle and clear square, respectively. AtsR previously incubated with H_2_O_2_ resulted in a mixture of native (dimer; clear circle) and oxidized (dimer; arrow) species. **B** Elution of native AtsR, oxidized AtsR, and reduced AtsR:C123SC187S from size exclusion column indicated by clear circle, arrow, and clear square, respectively. For calibration, a premixed protein molecular mass marker containing the following proteins was used: ribonuclease A (13,700 Da), carbonic anhydrase (29,000 Da), ovalbumin (44,000 Da), conalbumin (75,000 Da), and alcohol dehydrogenase (150,000 Da) (GE Healthcare, Piscataway, NJ). *V*_*o*_ was determined with blue dextran (2000 kDa). **C** Redox response of His_6_-AtsR, His_6_-AtsR:C123S, His_6_-AtsR:C187S, and His_6_-AtsR:C123SC187S detected by nonreducing SDS-PAGE. Proteins were incubated with or without H_2_O_2_ and CHP for 30 min, respectively, and then, when indicated, added with DTT and incubated for another 30 min. All samples were separated by 15% nonreducing SDS-PAGE and stained with Coomassie Brilliant Blue. M represented protein molecular mass marker. “−” expressed purified protein. **D** Quantification of free thiol levels in His_6_-AtsR, His_6_-AtsR:C123S, His_6_-AtsR:C178S, and His_6_-AtsR:C123SC178S. DTT−, H_2_O_2_−, and CHP-treated protein (20 µM) were mixed 5 mM with DTNB in 50 mM Tris-HCl buffer (pH 8.0), respectively, and the absorbance was monitored at 412 nm against a 2 mM DTNB solution as reference. These data were means of the values obtained from three independent assays. **E** Spectrophotometric analysis of NBD-labeled AtsR mutants. Proteins treated with DTT or without H_2_O_2_ were modified with NBD-Cl. The resulting proteins were analyzed spectrophotometrically at 200 to 600 nm

The upstream regions of the genes encoding AtsR homologs in *C. efficien*s, *C. crudilactis*, *C. callunae*, *C. deserti* were searched with the MEME software for a putative AtsR binding motif, and a 12-bp sequence motif similar to the one identified at the upstream region of *ncgl0887* gene in *C. glutamicum* was found. According to the alignment shown in Additional file 1: Fig. S9, an AtsR consensus sequence could be derived, which contained a perfect 5-bp inverted repeat: 5′-TGCAA-N_2_-TTGCA-3′. The fact that AtsR binding site was palindromic with 5 bp half-sites separated by 2 bp was consistent with AtsR binding as a homodimer, a conclusion supported by size-exclusion chromatography evidence for AtsR existing as a homodimer in its native form (Fig. 5A left plane and B). A binding motif of this type and this size in *Corynebacterium* was typical for TetR-type transcriptional regulators like TetR [20] or CamR [21].

Most members of the TetR family usually existed as homodimers and bound to target promoters as repressors [4]. In many cases, the function as transcriptional repressor correlated with a binding site within or downstream of the − 10/35 regions of the promoter. The typical TetR in Gram-negative bacteria negatively controlled the transcriptions of *tetA* and of its own gene (*tetR*) by binding to two identical operators separated by 11 bp that located at respective promoter region of *tetA* and of *tetR* and overlapped RNA polymerase binding site, thus affecting the binding of RNA polymerase to promoters [22, 23]. The Gram-positive *Staphylococcus aureus* QacR negatively controlled the *qacA* by binding to the motif that overlapped the region from − 6 to + 21 [24]. The repression thus prevented the transition of RNA polymerase-promoter complex into a productively transcribing state [24, 25]. RolR bound to a single operator *rolO*, which was located in the intragenic region of *rolR* and *rolHMD* and was not at the promoter region or overlapping with the transcription start site [8]. Due to the position of *rolO* distant to the transcription start sites of both genes (the overlapping + 66 to + 94 region for *rolHMD* and the + 40 to + 68 region for *rolR*), RolR repressed the transcription of *rolHMD* and of its own gene by a roadblock mechanism. In this study, AtsR bound to only one motif that was located at the upstream region of the *ncgl0887-atsR* operon, which was neither the promoter region of the *ncgl0887-atsR* operon nor overlapping with the transcription start site. Due to the position of the motif distant to the putative transcription start site of the *ncgl0887-atsR* operon (the overlapping + 93 to + 105 region), it is likely that AtsR repressed the transcription of the *ncgl0887-atsR* operon by a roadblock mechanism as PurR in *E. coli* [20] and RolR in *C. glutamicum* [8].

Members of the TetR family exerted their diverse regulatory mechanisms by using different forms of binding. Most TetR family transcription regulators reportedly bound to an about 15-bp operator as one homodimer containing a ~ 5-bp inverted repeat sequences [4]. For example, *C. glutamicum* BioQ dimer bound to a 13-bp palindromic motif TGAAC-N_3_-GTTAC [11]; *C. glutamicum* McbR bound to the consensus motif TAGAC-N_6_-GTCTA [7]. However, some TetR-type repressors bound to unusually long operator in more than two identical subunits-containing multimer. *S. aureus* QacR recognized and bound to the 28-bp operator IR1 as a pair of homodimers, consisting of a consensus motif [CTTATAGACCGATCGATCGGTCTATAAG] with a 14-bp perfect palindromic sequence [24]. Two homodimeric CgmR from *C. glutamicum* bound to a 32-bp operator site containing a 14-bp inverted repeat sequence [CGTAACTGTACCGA-N_4_-TCGTTACAGTTACG] [3]. *C. glutamicum* RolR bound to a 29-bp sequence of two perfect 5-bp palindromic repeats-REP1 (TGAAC-N_4_-GTTCA) and REP2 (TTCAT-N_2_-ATGAA) as a pair of homodimers [8]. *Mycobacterium tuberculosis* EthR bound to a 55-bp operator IG-55 cooperatively as a homo-octamer, including two copies of a long direct repeat (T-C-A-A-C-G-T/A-N-A-T-G-T-C-G-A) and two nearly perfect inverted repeats (T-A-A/G-T-G-T-C-G-A) [26]. Although CgmR, QacR, or RolR as a pair of homodimers bound to their operator in a similar manner, the three repressors exclusively recognized their inverted repeat sequences and the spacer sequence length between the binding sites. Thus, these previous studies probably suggested a direct relationship between the number of multimerization of TetR-type repressors and the length of the operator. In the current study, AtsR recognized the consensus motif [TGCAA-N_2_-TTGCA] with a 5-bp perfect palindromic sequence, which was nearly the same length to the operator (15 bp) of BioQ [11], McbR [7], and AcnR [9] from *C. glutamicum*. Since, in addition, we found that AtsR occurred as a homodimer in its native form, we proposed that AtsR bound to the operator as one homodimer.

### The ability of AtsR to bind the promoter region of the *ncgl0887***-***atsR* operon was inhibited by oxidant

**Fig. 6:**
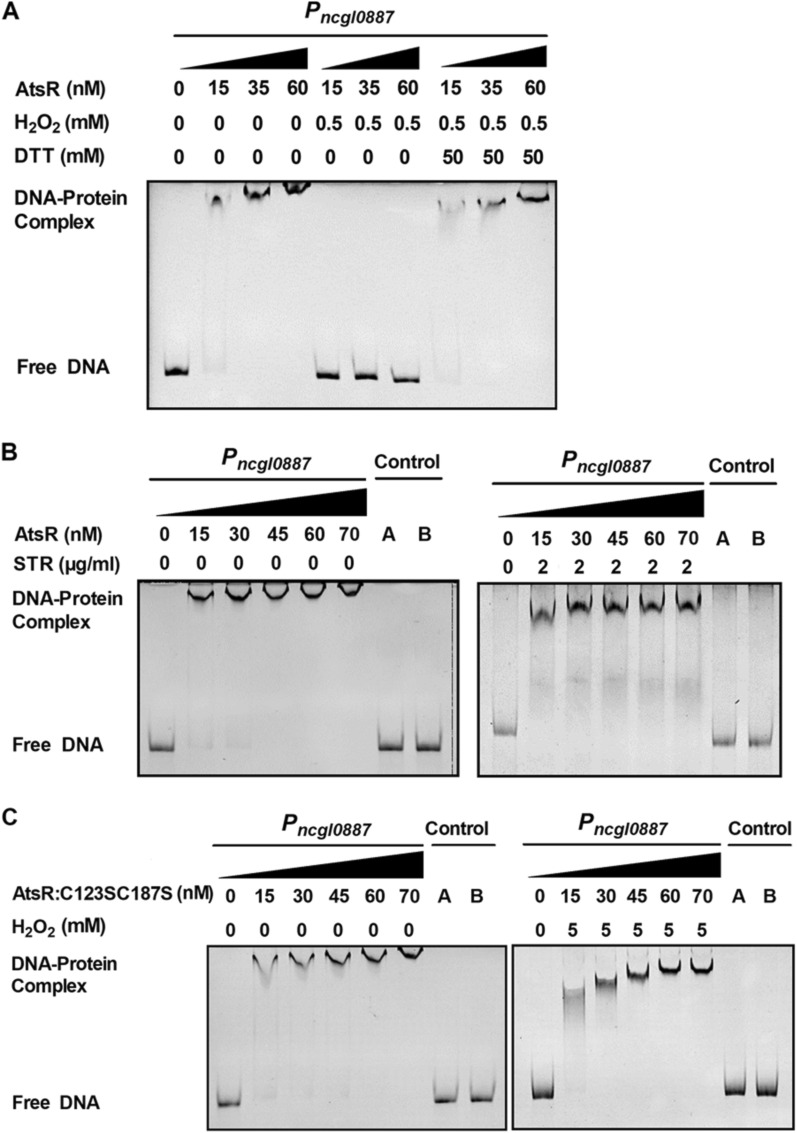
Reversible inhibition of the DNA binding activity of AtsR by H_2_O_2_ and role of cysteine residue.** A** Inhibition of the DNA binding activity of AtsR by H_2_O_2_ and reversal of the inhibition by DTT. AtsR was prepared in three different concentrations, and aliquots were taken for EMSAs (Control). Then H_2_O_2_ was added to the binding reaction mixture to a final concentration of 0.5 mM, and aliquots were taken for EMSA. In the next step DTT (a final concentration of 50 mM) was added to 0.5 mM H_2_O_2_-containing binding reaction mixture, and aliquots were taken for EMSAs. All aliquots were incubated in binding buffer, pH 8.0, with 40 ng *P*_*ncgl0887*_ and then separated on an 8% native polyacrylamide gel. **B** STR was added to the binding reaction mixture to a final concentration of 2 µg/ml, and the interaction between AtsR and *P*_*ncgl0887*_ was performed. **C** The interaction between the mutated derivatives AtsR:C123SC187S and *P*_*ncgl0887*_ in the absence (left panel) or presence (right left) of 5 mM H_2_O_2_. Results were obtained in three independent experiments, and data show one representative experiment done in triplicate

Interestingly, the binding of AtsR to *P*_*ncgl0887*_ was prevented by the addition of H_2_O_2_ (Fig. 6A). Importantly, the impaired DNA-binding activity of AtsR by H_2_O_2_ could be restored via the addition of an excess of the reducing agent DTT (50 mM), indicating that the effects of oxidation and reduction on the DNA-binding activity of AtsR were reversible. However, the addition of STR did not induce the dissociation of AtsR from *P*_*ncgl0887*_ (Fig. 6B), inconsistent with the finding that improved transcription of the *ncgl0887*-*atsR* operon under the STR-induced condition was mediated via AtsR in vivo (Figs. 1, 2, 3). Combined with the discovery that expression of the *ncgl0887*-*atsR* operon was affected by H_2_O_2_ (Figs. 1, 2, 3), we speculated that this was related to STR-mediated perturbation of the electron transfer chain, resulting in formation of ROS in vivo, which inactivated AtsR DNA-binding activity by the oxidation of cysteine residues [27, 28]. In fact, many studies have reported that the most potent xenobiotics, including oxidants, alkylating agents, antibiotics, and heavy metals, can generate ROS by redox cycling to produce oxidative stress inside bacteria [29, 30]. Thus, we speculated that AtsR did not directly sense ligands such as STR.

### Oxidant altered the conformation of AtsR via cysteine oxidation

Many stress-sensing regulators controlled the target gene expression by versatile posttranslational thiol-modifcations mechanisms, including the disulfdes-switch model (sulfenic acid, disulfde bond, and *S*-thiolation), Cysphosphorylation and Cys-alkylation [21, 31]. The amino acid sequence of AtsR showed that it contained two cysteine residues at positions 123 and 187 (Additional file 1: Fig. S1B). Many studies have reported that the most potent agents, including oxidants, alkylating agents, antibiotics, and heavy metals, could generate ROS by redox cycling to produce oxidative stress inside bacteria [29–32]. Thus, we thought it might also share a similar oxidation-sensing mechanism. As shown in Fig. 5C, nonreducing SDS-PAGE showed that purified His_6_-AtsR protein was monomeric with an apparent MW of approximately 27 kDa, in close agreement with the sum of its molecular mass (~ 21.5 kDa) deduced from amino acid sequence and His_6_ (about 5 kDa), while His_6_-AtsR incubated with H_2_O_2_ and CHP migrated as a band of approximately 54 kDa, as judged by its behavior on 15% nonreducing SDS-PAGE, which corresponded to His_6_-AtsR in its dimeric form. The dimeric formation was reversed by an excess of DTT (Fig. 5C). These results suggested that there was the formation of Cys123-Cys187 intermolecular disulfide bonds between two monomeric His_6_-AtsR protein under oxidants treatment. To confirm the speculation, mutants, His_6_-AtsR:C123S, His_6_-AtsR:C187S, and His_6_-AtsR:C123SC187S, in which cysteine residue was replaced with Ser, were constructed and used. As shown in Fig. 5C, treatment of H_2_O_2_ and CHP did not result in any dimer formation of His_6_-AtsR:C123S, His_6_-AtsR:C187S, and His_6_-AtsR:C123SC187S. The result was further confirmed by measuring the thiol content of H_2_O_2_- and CHP-treated proteins with the DTNB assay. DTNB assay showed the DTT-treated His_6_-AtsR contained two thiols per monomer, as the thiol content of DTT-treated His_6_-AtsR was 1.795 ± 0.075 thiol groups per monomer. However, H_2_O_2_- and CHP-treated His_6_-AtsR were found to contain 0.110 ± 0.05 and 0.139 ± 0.08 thiol groups per monomer, indicating that there was no thiol in His_6_-AtsR upon oxidant treatment (Fig. 5D). The His_6_-AtsR:C123S showed one thiol per monomer before and after H_2_O_2_ treatment. However, the His_6_-AtsR:C187S under H_2_O_2_ treatment lost one thiol group, compared to the thiol content of DTT-treated states. These results indicated that Cys123 was the peroxidatic Cys (C_P_) residue that was more susceptible to oxidation and was easily oxidized to form sulfenic acid (Cys-SOH), but Cys187 might be the resolving Cys residues (C_R_). To investigate the possibility, NBD-Cl assay was performed. NBD-Cl exclusively reacted with thiol groups and sulfenic acids, but not with sulfinic or sulfonic forms. The covalent attachment of NBD-Cl generated an absorption peak at about 420 nm upon reaction with thiol groups, whereas it peaked at about 347 nm upon reaction with sulfenic acids [33]. Following the reaction with NBD-Cl, the absorption spectra of the His_6_-AtsR:C123S protein was unchanged before or after exposure to H_2_O_2_ (Fig. 5E), exhibiting only the 420 nm peak. However, His_6_-AtsR:C187S protein with H_2_O_2_ treatment showed soret band at 347 and 420 nm, indicating that the reaction of NBD-Cl with sulfenic acids and free thiol groups existed at the same time. Therefore, we speculated that Cys123 was partly oxidized to sulfenic acid form (Fig. 5E). These data indicated that Cys123 in a subunit of AtsR dimer was oxidized by H_2_O_2_ to form Cys-SOH, and then directly reacted with Cys178 in another subunit of AtsR dimer to generate intermolecular disulfide bond, which was similar to the observation of *C. glutamicum* CosR that was a reversible disulfide bonding between the two subunits of the protein under oxidation treatment [34].

Interestingly, size exclusion chromatography showed that native AtsR protein treated CHP was eluted as two separate species, the main one with *M*w ≈ 41.7 kDa and another with *Mw* ≈ 44.7 kDa (Fig. 5A middle plane and B), suggested that CHP-treated AtsR protein still existed as dimer and oxidized AtsR protein formed covalently crosslinked dimer. Beacuse the main one with *M*w ≈ 41.7 kDa (corresponding to oxidized AtsR) seemed to form the Cys123-Cys187 intermolecular disulfide bond between two subunits in AtsR dimer, but the minor one (corresponding to reduced AtsR) with *Mw* ≈ 44.7 kDa did not. Further, size exclusion chromatography showed His-tag-free AtsR:C123SC187S proteins also exhibited homodimer (Fig. 5A right plane and B). The above results, combined with a phenomenon reduced AtsR existed as dimer (Additional file 1: Fig. S10A), indicated native AtsR existed as homodimer by joining two identical subunits together with noncovalent bonds, but Cys123 in a subunit of the AtsR dimer formed intermolecular disulfide bonds with Cys187 in another subunit of the AtsR dimer upon exposure to oxidants.

In addition, analysis of transcription levels revealed that Δ*atsR*(pXMJ19-*atsR:C123SC187S*) strains exhibited lower expression of *ncgl0887*-*atsR* operon than those in the WT(pXMJ19) and Δ*atsR*(pXMJ19-*atsR*) strains under low concentration of STR and H_2_O_2_, indicating that Cys123 and Cys187 played roles in the dissociation of AtsR from the promoter under low concentration of agents (Figs. 1, 2, 3); EMSA experiment also exhibited AtsR:C123SC187S obviously remained binding to the promoter DNA in the presence or absence of H_2_O_2_ (Fig. 6C). Although its affinity constant for *P*_*ncgl0887*_ (*K*_*D*_ ≈ 42) was slightly higher than that of AtsR, AtsR:C123SC187S behaved high similarly to AtsR without H_2_O_2_ condition (Additional file 1: Fig. S8B). These results imply that oxidation of Cys123 and Cys187 were important for inhibition of DNA binding by H_2_O_2_. Therefore, we speculated that AtsR regulated genes involved in stress response through a thiol-based mechanism. This result suggested that AtsR formed Cys123-Cys187 intermolecular disulfide bonds between two monomers in dimeric AtsR, as a result of which it dissociated from the target DNA sequence, thereby activating the target regulon. These results further indicated that H_2_O_2_ caused a structural change in dimeric AtsR and that Cys123 and C187 were responsible for the morphological changes in dimeric AtsR observed under H_2_O_2_ treatment.

## Conclusions

In this study, we investigated how AtsR regulated the transcription of the *ncgl0887*-*atsR* operon and *ncgl0884* in *C. glutamicum*. *ncgl0884* and *ncgl0887* encoded a multidrug efflux pump protein and a resistance, nodulation and cell division (RND) superfamily drug exporter, respectively. The results showed that AtsR directly repressed the transcription of the *ncgl0887*-*atsR* operon by binding to a 12-bp sequences. However, AtsR indirectly controlled the transcription of *ncgl0884*. The 12-bp motif was the sole binding site for AtsR, and it located in the upstream region of the *ncgl0887*-*atsR* operon. The binding of AtsR was affected by H_2_O_2_. H_2_O_2_ was able to dissociate AtsR from the promoter DNA, thus derepressing the transcription of the *ncgl0887*-*atsR* operon in *C. glutamicum*. When AtsR was overexpressed, the tolerance of *C. glutamicum* RES167 strain to several toxic compounds was obviously decreased, and the effect of AtsR overexpression on the drug tolerance of the *C. glutamicum* RES167 strain might be mediated by NCgl0884 and NCgl0887. Together, the results revealed that AtsR was a key TetR-type redox stress-responsive transcriptional repressor and sensed stress.

## Methods

### Bacterial strains and growth conditions

Bacterial strains and plasmids used in this study were listed in Additional file 1: Table S1. *Escherichia coli* and *C. glutamicum* strains were grown in Luria-Bertani (LB) medium or minimal medium (MM) with appropriate antibiotics as previously reported [35]. To produce mutant of a gene in *C. glutamicum*, brain-heart infusion broth medium containing 0.5 M sorbitol (BHIS) was used [36]. The *C. glutamicum* RES167 strain (restriction-deficient variant derived from the ATCC 13,032 type) was the parent of all derivatives used in this study. In-frame deletions of *atsR*, *ncgl0884* and *ncgl0887* were generated as described [37]. Chromosomal deletion of *atsR*, *ncgl0884*, or *ncgl0887* was further confirmed by DNA sequencing. For complementation or overexpression, the pXMJ19 derivatives were transformed into the corresponding mutants or the *C. glutamicum* RES167 parent strain by electroporation [37]. 0.5 mM isopropyl β-D-thiogalactopyranoside (IPTG) (Sigma-Aldrich) was added into medium to induce the expression of the target gene on the pXMJ19 derivatives. Antibiotics were added at the following concentrations: Kanamycin (KAN), 50 µg/ml for *E. coli* and 25 µg/ml for *C. glutamicum*; nalidixic acid (NAL), 40 µg/ml for *C. glutamicum*; chloramphenicol (CHL), 20 µg/ml for *E. coli* and 10 µg/ml for *C. glutamicum*.

### Plasmid construction

Primers used in this study were listed in Additional file 1: Table S2. The genes encoding *C. glutamicum ncgl0884*, *atsR* (*ncgl0886*), and *ncgl0887* were amplified by polymerase chain reaction (PCR). These DNA fragments were digested and inserted into pET28a, pET28a-SUMO, and pXMJ19 to obtain pET28a-*atsR*, pET28a-SUMO-*atsR*, pET28a-*ncgl0887*, pXMJ19-*ncgl0884*, pXMJ19-*atsR*, and pXMJ19-*ncgl0887*, respectively.

The knockout plasmids pK18*mobsacB*-Δ*ncgl0884*, pK18*mobsacB*-Δ*atsR* and pK18*mobsacB*-Δ*ncgl0887*, used to construct the in-frame deletion mutants of *C. glutamicum* RES167, were made by overlapping PCR [38].

The *lacZY* fusion reporter vectors pK18*mobsacB-P*_*ncgl0884*_::*lacZY* and pK18*mobsacB- P*_*ncgl0887*_::*lacZY* were made by the fusion of the promoter DNA fragments of *ncgl0884* (814-bp, from − 799 to + 15 bp) and *the ncgl0887-atsR* operon (768-bp, from − 753 to + 15 bp) (distance was with respect to the start codon of ORF of *ncgl0884* or *ncgl0887*) to the *lacZY* reporter gene via overlapping PCR [38].

For obtaining pK18*mobsacB-P*_*ncgl0887M*_::*lacZY*, 768-bp promoter DNA containing mutated sequence of the identified AtsR binding site (*P*_*ncgl0887M*_) was first constructed by means of overlap PCR [38]. Briefly, two rounds of PCR are used. In the first round of PCR, primer pairs *P*_*ncgl0887*_-F/*P*_*ncgl0887*_-mutation-R and *P*_*ncgl0887*_-mutation-F/Oncgl0887-R were used to amplify segments 1 and 2, respectively. The second round of PCR was carried out using *P*_*ncgl0887*_-F/*P*_*ncgl0887*_-R as primers and fragment 1 and fragment 2 as templates to get the *P*_*ncgl0887M*_ DNA fragment. Mutated sequence as *P*_*ncgl0887*_-mutation-F primer was shown in blue below the promoter sequence (Additional file 1: Fig. S2A). Then, the resulting 768-bp *P*_*ncgl0887M*_ DNA fragment was fused to a *lacZY* reporter gene using *P*_*ncgl0887*_-F/lacZY-R as primers. Finally, *P*_*ncgl0887M*_::*lacZY* was inserted into pK18*mobsacB.*

The fidelity of all constructs was confirmed by DNA sequencing (Sangon Biotech, Shanghai, China).

### Overexpression and purification of recombinant protein

To express and purify His_6_-tagged proteins, pET28a derivatives were transformed into *E. coli* BL21-Star (DE3) host cells (Stratagene, La Jolla, CA). Recombinant proteins were purified with the His·Bind Ni-NTA resin (Novagen, Madison, WI) according to manufacturer’s instructions. Although His-tagged protein can remain soluble at 4 ℃ in a shorter period of time, it showed a stronger tendency to aggregation and precipitation after stored at -80 ℃. Therefore, the tag was cleaved off for experiments in which it was crucial to keep the protein stable over some time to get reproducible results, such as EMSA, the determination of the apparent *K*_*D*_ values, or the reversibility of the inhibition of DNA binding by agents. The protein without His-tag was most stable when stored frozen at − 80 °C (Additional file 1: Fig. S10B). Cleavage of the His_6_-SUMO tag was performed by adding PUP1 (0.04 mg PUP1 protein was used to cut 1 mg His_6_-SUMO-AtsR or His_6_-SUMO-AtsR:C123S178S) and incubation at 4 °C overnight. Ni-NTA resin was used to remove the cleaved tag and uncleaved protein from the His_6_-SUMO-free protein. The BL21-Star (DE3)(pET28a-*pup1*) was kindly provided by Tietao Wang at Key Laboratory of Resources Biology and Biotechnology in Western China, Ministry of Education, College of Life Sciences, Northwest University, Xi’an, China. Protein concentrations were determined using the Bradford assay [39].

### Electrophoretic mobility shift assay (EMSA)

The binding of AtsR to the promoter of *ncgl0884* gene or the *ncgl0887*-*atsR* operon was performed using the method of Wang et al. [40]. Briefly, increasing concentrations of purified AtsR (0–70 nM) were incubated with 232-bp promoter DNA of the *ncgl0887*-*atsR* operon (*P*_*ncgl0887*_, 40 ng) that contained the predicted AtsR binding site and was amplified from the sequence (− 252 to − 21 relative to the GTG start codon of *ncgl0877* ORF) using primer pair E_ncgl0887_-F/E _ncgl0887_-R or 205-bp promoter DNA of *ncgl0884* gene (*P*_*ncgl0884*_, 40 ng) that contained the predicted AtsR binding site and was amplified from the sequence (− 185 to + 20 relative to the ATG start codon of the *ncgl0884* ORF) using primer pair E_ncgl0884_-F/E_ncgl0884_-R in a total reaction volume of 20 µl. 232-bp and 205-bp fragments from the *ncgl0887* and *ncgl0884* coding region amplified with primers Control-F1/Control-R1 and Control-F2/Control-R2 instead of *P*_*ncgl0887*_ and *P*_*ncgl0884*_, respectively, and bovine serum albumin (BSA) instead of AtsR were used as negative controls. 232-bp mutant EMSA promoter DNA fragment (232-bp *P*_*ncgl0887M*_) containing the mutated sequence of the predicted AtsR-binding site and having the same nucleotide sequence as 232-bp *P*_*ncgl0887*_ except for mutation sites was directly synthesized by Shanghai Biotechnology Co., Ltd. The mutated sequence was shown in blue below the promoter sequence in Additional file 1: Fig. S2A. The binding reaction buffer contained 10 mM Tris-HCl (pH 7.4), 5 mM MgCl_2_, 50 mM KCl, 5% (v/v) glycerol, 0.1%(v/v) Nonidet P 40 (NP40), 1 µg poly(dI:dC), 1 mM dithiothreitol (DTT). The binding reaction mixtures were incubated at room temperature for 30 min and then loaded onto 8% native PAGE made with 10 mM Tris buffer containing 50 mM KCl, 5 mM MgCl_2_ and 10% glycero1 in 0.5 × TBE electrophoresis buffer [50 mM Tris base, 41.5 mM boric acid (pH 8.0), 10 mM Na_2_EDTA.H_2_O]. Electrophoresis was performed at 100 V for 2 h on ice using 1 × TBE (89 mM Tris base, 89 mM boric acid, 2 mM EDTA) as buffer. The gel was subsequently stained with a 10,000-fold diluted SYBR Gold nucleic acid staining solution (Molecular Probes) for 30 min. The DNA bands were visualized with UV light at 254 nm. For the determination of apparent *K*_*D*_ values, photographed were quantified using ImageQuant software (GE Healthcare), and the percentage of shifted DNA was calculated. These values were plotted against the AtsR concentration in log_10_ scale, and a sigmoidal fit was performed using GraphPad Prism software (GraphPad Software, San Diego California USA), considering the error bars as well as 0 and 100% shifted DNA as asymptotes, the turning point of the curve was defined as the apparent *K*_*D*_ value. All determinations were performed in triplicate.

Three mutated fragments in Fig. 4F were first directly synthesized by Shanghai Biotechnology Co., Ltd., each with two or three nucleotides exchanged, and tested again by EMSAs with excess AtsR.

The loss of binding due to antibiotic and toxic compound was tested as follows. Streptomycin (STR, a final concentration of 2 µg/ml) and hydrogen peroxide (H_2_O_2_, a final concentration of 0.5 or 5 mM) were added to the binding buffer containing different concentrations of purified AtsR and 40 ng *P*_*ncgl0887*_ for EMSA, respectively. The binding buffer was incubated at room temperature for 30 min and then separated on an 8% nondenaturing PAGE and the gel was stained using SYBR Gold nucleic acid staining solution.

### Construction of chromosomal fusion reporter strains and β-galactosidase assay

The *lacZY* fusion reporter plasmids pK18*mobsacB*-*P*_*ncgl0884*_::*lacZY*, pK18*mobsacB*-*P*_*ncgl0887*_::*lacZY*, and pK18*mobsacB*-*P*_*ncgl0887M*_::*lacZY* were transformed into corresponding *C. glutamicum* by electroporation. The transformants were selected by plating on LB agar plates containing 40 µg/ml NAL, 25 µg/ml KAN, and 10 µg/ml CHL [14]. The resulting strains were grown in LB medium to an optical density at 600 nm of 0.6–0.7 and then treated with different reagents of various concentrations at 30 °C for 30 min. β-galactosidase activities were assayed with *o*-Nitrophenyl-β-d-galactopyranoside (ONPG) as the substrate [41]. All β-galactosidase experiments were performed with at least three independent biological replicates.

### Quantitative real-time polymerase chain reaction (qRT-PCR) analysis

Total RNA was isolated from exponentially growing WT(pXMJ19), Δ*atsR*(pXMJ19) and Δ*atsR*(pXMJ19-*atsR*) strains exposed to different toxic agents of indicated concentrations for 30 min using the RNeasy Mini Kit (Qiagen, Hilden, Germany) along with the DNase I Kit (Sigma-Aldrich, Taufkirchen, Germany). Purified RNA was reverse-transcribed with random 9-mer primers and MLV reverse transcriptase (TaKaRa, Dalian, China). Quantitative RT-PCR analysis (7500 Fast Real-Time PCR; Applied Biosystems, Foster City, CA, USA) was performed as described previously [42]. To obtain standardization of results, the relative abundance of 16 S rRNA was used as the internal standard.

### Antimicrobial susceptibility analysis

Minimal inhibitory concentrations (MICs) were determined using a standard 2-fold serial dilution format on LB broth medium [43]. Briefly, agents were serially diluted (0.5 ×) in LB broth (1 ml) and 10 µl cells grown at 30 °C in LB medium to the stationary phase were transferred to 1 ml LB broth with and without addition of various concentrations of antibiotics and toxic compounds. After 1 to 2 days of incubation at 30 °C, the tubes were checked for growth.

### Western blot analysis

Western blot analysis was performed as described previously [44]. Primary antibodies at 4 °C overnight: anti-NCgl0887 rabbit polyclonal antibody, 1:1000; anti-cytosolic RNA polymerase α (α-RNAP), 1:5000 (BioLegend Way, San Diego, California, USA). The α-RNAP was used as a loading control as our previous studies [14]. The anti-NCgl0887 rabbit polyclonal antibodies were generated and affinity-purified according to the method described previously [45]. The density of bands on Western blots was quantified by Image Lab (Bio-Rad, California, USA).

### Size exclusion chromatography

The size of purified His_6_-AtsR was estimated by gel filtration on Superdex 200 10/300 GL column (GE Healthcare, Piscataway, NJ, USA) using a buffer (50 mM potassium phosphate [pH 7.4], 0.15 M NaCl) with a gel filtration calibration kit (low molecular weight; GE, UK). The calibration curve was plotted by use of the *K*_av_ versus the logarithm of the molecular weight.

### Quantitative analysis of sulfhydryl groups

Free thiol content of AtsR was measured by using 5,5′-dithio-bis (2-nitrobenzoic acid) (DTNB) [46].

### Analysis of sulfenic acid formation

The formation of sulfenic acid was measured by the assays of DTT-treated or H_2_O_2_-treated proteins labeled with 4-chloro-7-nitrobenzofurazan (NBD-Cl) [33].

### The redox state of AtsR

The redox state of 20 µM His_6_-AtsR was analyzed by incubating the proteins with 50 mM DTT, 5 mM H_2_O_2_ and 4 mM cumene hydroperoxide (CHP) 30 min before separating on nonreducing 15% SDS-PAGE [34]. Moreover, H_2_O_2_- and CHP-His_6_-AtsR were added with 50 mM DTT and then incubated for another 30 min before separating on nonreducing 15% SDS-PAGE. For nonreducing conditions, the loading buffer [250 mM Tris-HCl (pH6.8), 0.5% bromophenol blue (BPB), and 50% (v/v) glycerol] was added to treated protein samples. All the samples were boiled for 5 min prior to electrophoresis and then stained with Coomassie Brilliant Blue (CBB). The experiment was performed in triplicate.

### Statistical analysis

Statistical analyses of survival rate, transcription level and protein level were determined with paired two-tailed Student’s *t*-test. GraphPad Prism Software was used to carry out statistical analyses (GraphPad Software, San Diego, California,  USA).

**Table 1:** The role of AtsR, NCgl0887 and NCgl0884 in stress response

Compound	MIC									
	WT (pXMJ19)	WT (pXMJ19-*atsR*)	Δ*atsR* (pXMJ19)	Δ*atsR* (pXMJ19-*atsR*)	Δ*ncgl0884* (pXMJ19)	Δ*ncgl0884* (pXMJ19-*ncgl0884*)	Δ*ncgl0887* (pXMJ19)	Δ*ncgl0887* (pXMJ19-*ncgl0887*)	Δ*ncgl0884* (pXMJ19-*atsR*)	Δ*ncgl0887* (pXMJ19-*atsR*)
RIF(µg/ml)	0.00125	0.0025	0.00125	0.00125	0.0025	0.00125	0.00125	0.00125	0.005	0.00125
ERY (µg/ml)	1	0.125	1	1	0.125	1	1	1	0.125	1
SPE (µg/ml)	30	1.875	30	30	15	30	7.5	30	15	7.5
PEN (µg/ml)	350	43.75–87.5	350	350	87.5	350	43.75	350	87.5	43.75
NEO (µg/ml)	0.7	0.175	0.7	0.7	0.35	0.7	0.175	0.7	0.35	0.175
VAN (µg/l)	0.7	0.175	0.7	0.7	0.7	0.7	0.7	0.7	0.7	0.7
NOR (µg/ml)	1.25	0.078	1.25	1.25	0.625	1.25	0.3125	1.25	0.625	0.3125
STR (µg/ml)	1.25	0.156	1.25	1.25	1.25	1.25	0.3125	1.25	1.25	0.3125
GEN (µg/ml)	0.6	0.075	0.6	0.6	0.15	0.6	0.075	0.6	0.075	0.075
LIN (µg/ml)	9	1.125	9	9	1.5	9	1.5	9	1.55	1.5
H_2_O_2_ (mM)	110	13.75	100	110	27.5	110	55	110	27.5	55
Diamide (mM)	3	0.375	3	3	0.75	3	0.375	3	0.75	0.375
CDNB (µM)	7	7	7	7	7	7	7	7	7	7
IAM (µM)	300	150	300	300	300	300	300	300	300	300
EB (µM)	8	0.5–1	8	8	2	8	4	8	2	4
SDS (µg/ml)	200	50	200	200	200	200	50	200	50	50
CHP (mM)	0.69	0.1725	0.62	0.69	0.345	0.69	0.345	0.69	0.345	0.345
BZK (mM)	0.5	0.125–0.25	0.5	0.5	0.25	0.5	0.125	0.5	0.25	0.125
MEN (mM)	0.72	0.18–0.36	0.6	0.72	0.36	0.72	0.36	0.72	0.36	0.36
CdCl_2_ (µM)	60	30	60	60	30	60	60	60	30	60
CuCl_2_ (µM)	6	3	6	6	3	6	6	6	3	6
K_2_CrO_7_ (mM)	0.7	0.35	0.7	0.7	0.35	0.7	0.7	0.7	0.7	0.7
NiSO_4_ (mM)	2.5	0.625	2.5	2.5	0.625	2.5	2.5	2.5	2.5	2.5

^a^ MIC determination experiments were repeated at least three times. RIF, Rifampin; ERY, erythromycin; SPE, spectinomycin; PEN, penicillin; NEO, neomycin; VAN, vancomycin; NOR, norfloxacin; STR, streptomycin; GEN, gentamycin; LIN, lincomycin; H_2_O_2_, hydrogen peroxide; CHP, cumene hydrogen peroxide; CDNB, 1-chloro-2,4-dinitrobenzene; IAM, iodoacetamide; EB, ethidium bromide; SDS, sodium dodecyl sulfate; BZK, benzalkonium; MEN, menadione; CdCl_2_, cadmium chloride; CuCl_2_, copper chloride; K_2_CrO_7_, potassium dichromate; NiSO_4_, nickel sulfate

## Supplementary information

**Additional file 1. Table S1.** Bacterial strains and plasmids used in this study. **Table S2.** Primers used in this study. **Fig. S1** Multiple sequence alignment. **Fig. S2** Detailed genetic maps. **Fig. S3**. Assays for the *ncgl0887*-*atsR* co-transcription by reverse transcription PCR. **Fig. S4** Growth curves of the WT(pXMJ19) strain (the *C. glutamicum* RES167 parental strain transformed with the empty plasmid pXMJ19), the Δ*atsR*(pXMJ19) mutant (the *atsR* deletion mutant expressing pXMJ19), the WT(pXMJ19-*atsR*) strain (*C. glutamicum* RES167 parental strain expressing the wide-type (WT) *atsR* gene in the shuttle vector pXMJ19), Δ*ncgl0884*(pXMJ19) mutant (the *ncgl0884* deletion mutant expressing pXMJ19), and Δ*ncgl0887*(pXMJ19) mutant (the *ncgl0884* deletion mutant expressing pXMJ19) under normal conditions. **Fig. S5** 86-bp *atsR* transcript (from the translational start codon (ATG) of *atsR* gene to 86th nucleotide) was amplified from the remaining *atsR* ORF (open reading frame) in Δ*atsR* mutant with primers QatsR-F and QatsR-R. **Fig. S6** The NCgl0887 was examined in *C. glutamicum*. **Fig. S7** Negative regulation of *ncgl0884* expression by AtsR. **Fig. S8** Determination of the apparent *K*_*D*_ values of AtsR and AtsR:C123SC187S for *P*_*ncgl0887*_. **Fig. S9** Sequence of the promoter region of *C. glutamicum* *ncgl0887*-*atsR* operon aligned to putative promoter regions from other *Corynebacterium* species. **Fig. S10** Purification of AtsR.

## Data Availability

All the data generated or analyzed during this study are included in the manuscript and its Additional files.

## Abbreviations

TetR: Tetracycline repressor
ROS: Reactive oxygen species
DTNB: 5,5′-dithio-bis(2-nitrobenzoic acid)
EMSA: Electrophoretic mobility shift assay
CHP: Cumene hydroperoxide
H_2_O_2_: Hydrogen peroxide
DTT: Dithiothreitol
CBB: Coomassie Brilliant Blue
β-ME: β-Mercaptoethanol
ONPG: *O*-Nitrophenyl-β-d-Galactopyranoside
IPTG: Isopropyl β-d-1-thiogalactopyranoside
PVDF: Polyvinylidene fluoride
α-RNAP: Anti-cytosolic RNA polymerase α
