# Supplementary material for: The TetR-type regulator AtsR is involved in multidrug response in Corynebacterium glutamicum
Source: Microb Cell Fact. 2022 Jun 21;21:123. doi: 10.1186/s12934-022-01850-0 (PMC9210681; doi:10.1186/s12934-022-01850-0)
Supplement: Supplementary file 1 — Additional file 1. Table S1. Bacterial strains and plasmids used in this study. Table S2. Primers used in this study. Fig. S1 Multiple sequence alignment. Fig. S2 Detailed genetic maps. Fig. S3. Assays for the ncgl0887-atsR co-transcription by reverse transcription PCR. Fig. S4 Growth curves of the WT(pXMJ19) strain (the C. glutamicum RES167 parental strain transformed with the empty plasmid pXMJ19), the ΔatsR(pXMJ19) mutant (the atsR deletion mutant expressing pXMJ19), the WT(pXMJ19-atsR) strain (C. glutamicum RES167 parental strain expressing the wide-type (WT) atsR gene in the shuttle vector pXMJ19), Δncgl0884(pXMJ19) mutant (the ncgl0884 deletion mutant expressing pXMJ19), and Δncgl0887(pXMJ19) mutant (the ncgl0884 deletion mutant expressing pXMJ19) under normal conditions. Fig. S5 86-bp atsR transcript (from the translational start codon (ATG) of atsR gene to 86th nucleotide) was amplified from the remaining atsR ORF (open reading frame) in ΔatsR mutant with primers QatsR-F and QatsR-R. Fig. S6 The NCgl0887 was examined in C. glutamicum. Fig. S7 Negative regulation of ncgl0884 expression by AtsR. Fig. S8 Determination of the apparent KD values of AtsR and AtsR:C123SC187S for Pncgl0887. Fig. S9 Sequence of the promoter region of C. glutamicum ncgl0887-atsR operon aligned to putative promoter regions from other Corynebacterium species. Fig. S10 Purification of AtsR. [file 12934_2022_1850_MOESM1_ESM.docx]

**Supplemental Data**

**The TetR-Type regulator AtsR is involved in multidrug response in *Corynebacterium glutamicum***

Tao Su^1#*^, Chengchuan Che^1#^, Jiyu Han^1^,Yuying Zhao^1^, Zihan Zhang^1^, Guangdi An^1^, Meiru Si^1^, Can Chen^2*^

^1^College of Life Sciences, Qufu Normal University, Qufu, Shandong 273165, China;

^2^ Key Laboratory of Plant Genetics and Molecular Breeding, College of Life Science and Agronomy, Zhoukou Normal University, Zhoukou, Henan 466001, China

**Running title:** Multidrug-sensing mechanism of AtsR

**^#^** These authors contributed equally to this work.

**^*^** Corresponding authors:

Tao Su, Can Chen

E-mail [vincenttao2@163.com](mailto:vincenttao2@163.com); chenc02@126.com

Tel: 86-15563718633;86-18736207816

**Table S1. Bacterial strains and plasmids used in this study.**

| **Strains or plasmids** | **Relevant genotype description** | **References** |
| --- | --- | --- |
| **Strains** | | |
| ***Corynebacterium glutamicum*** | | |
| RES167 | Restriction-deficient mutant of ATCC13032, Δ(*cglIM-cglIR-cglIIR*) | [1] |
| Δ*atsR* | *atsR* deleted in RES167 | This study |
| Δ*ncgl0884* | *ncgl0884* deleted in RES167 | This study |
| Δ*ncgl0887* | *ncgl0887* deleted in RES167 | This study |
| WT(pXMJ19) | *C. glutamicum* RES167 parental strain (WT) transformed with the empty plasmid pXMJ19 | This study |
| Δ*atsR*(pXMJ19) | Δ*atsR* transformed with empty plasmid pXMJ19 | This study |
| Δ*atsR*(pXMJ19-*atsR*) | Δ*atsR* transformed with plasmid pXMJ19-*atsR* | This study |
| WT(pXMJ19-*atsR*) | *C. glutamicum* RES167 parental strain transformed with plasmid pXMJ19-*atsR* | This study |
| Δ*atsR*(pXMJ19-*atsR:C123SC187S*) | The Δ*atsR* mutant expressed pXMJ19-*atsR:C123SC187S* | This study |
| ***E. coli*** | | |
| BL21(DE3) | *E. coli* expression host, *hsdS gal* (*λc*I*ts*857 *ind-l* *Sam7 nin-*5 *lac UV5-*T7 gene 1) | Novagen |
| JM109 | *recA1 supE44 endA1 hsdR17 gyrA96 relA1 thi* Δ(*lac-proAB*)F′(*traD36 proABlacI*^q^ *lacΔZM15*) | Stratagene |
| **Plasmids** | | |
| pK18*mobsacB* | Suicide plasmid carrying *sacB* for selecting double crossover in *C. glutamicum*, Km^r^ | [2] |
| pK18*mobsacB-*Δ*atsR* | Construct used for in-frame deletion of *atsR* | This study |
| pK18*mobsacB-*Δ*ncgl0884* | Construct used for in-frame deletion of *ncgl0884* | This study |
| pK18*mobsacB-*Δ*ncgl0887* | Construct used for in-frame deletion of *ncgl0887* | This study |
| pK18*mobsacB-P_ncgl0887_::lacZY* | *P_ncgl0887_::lacZY* fusion in pK18*mobsacB* | This study |
| pK18*mobsacB-P_ncgl0884_::lacZY* | *P_ncgl0884_::lacZY* fusion in pK18*mobsacB* | This study |
| pXMJ19 | Shuttle vector (*P_tac_ lacI^q^ pBL1 oriV_C. glutamicum_* pK18 *oriV_E. coli_*) | [3] |
| pXMJ19-*atsR* | *atsR* cloned into pXMJ19 for complementation | This study |
| pXMJ19-*atsR:C123SC187S* | *atsR:C123SC187S* cloned into pXMJ19 for complementation |  |
| pXMJ19-*ncgl0884* | *ncgl0884* cloned into pXMJ19 for complementation | This study |
| pXMJ19-*ncgl0887* | *ncgl0887* cloned into pXMJ19 for complementation | This study |
| pET28a | Expression vector with N-terminal hexahistidine affinity tag | Novagen |
| pET28a-SUMO | Expression vector with N-terminal hexahistidine affinity tag and SUMO | This study |
| pET28a*-pup1* | *pup1* in pET28a | This study |
| pET28a*-atsR* | *atsR* in pET28a | This study |
| pET28a*-atsR:C123SC187S* | *atsR:C123SC187S* in pET28a | This study |
| pET28a*-*SUMO*-atsR* | *atsR* in pET28a-SUMO | This study |
| pET28a*-ncgl0887* | *ncgl0887* in pET28a | This study |
| pET28a-SUMO-*atsR:C123SC187S* | *atsR:C123SC187S* in pET28a-SUMO | This study |

**Additional References**

1. Tauch A, Kirchner O, Löffler B, Götker S, Pühler A, Kalinowski J. Efficient electrotransformation of C*orynebacterium diphtheriae* with a mini-replicon derived from the *Corynebacterium glutamicum* plasmid pGA1. Curr. Microbiol. 2002; 45 : 362-367.
2. Jakoby M, Ngouoto-Nkili CE, Burkovski A. Construction and application of new *Corynebacterium glutamicum* vectors. Biotechnol Tech.1999;113: 437-441.
3. Karimova G, Pidoux J, Ullmann A, Ladant D. A bacterial two-hybrid system based on a reconstituted signal transduction pathway. [Proc Natl Acad Sci U S A](http://www.ncbi.nlm.nih.gov/pubmed/9576956). 1998;95:5752-5756.

**Table S2.** Primers used in this study.

| **Primers** | **5’-3’ sequence** |  |
| --- | --- | --- |
| *atsR*_com_-F1 | CGCGGATCCAAAGGAGGACAACCGTGTCGGGGCTTAGAGAAAC (*Bam*HI) | For cloning *atsR* wild type and mutants into pXMJ19 |
| *atsR*_com_-R1 | CAAGAATTCTTATTTCTTAATCCAGGCGC (*Eco*RI) |  |
| OatsR-F | CAAGAATTCATGGGTCGCGGATCCGAATTCGTGTCGG  GGCTTAGAGAAACAA (*Eco*RI) | For cloning *atsR* wild type into pET28a |
| OatsR-R | CCGCTCGAGTTATTTCTTAATCCAGGCGCATGC (*Xho*I) |  |
| OatsR-F1 | CGCGGATCCGTGTCGGGGCTTAGAGAAACAA (*Bam*HI) | For cloning *atsR* wild type and mutant into pET28a-SUMO |
| DatsR-F1 | CAAGAATTCCTATGACATGATTACGAATTCCAGAATTTGATGCGATCGATGA (*Eco*RI) | To generate pK18*mobsacB-*Δ*atsR* |
| DatsR-R1 | GGATCAATGCGACTCCATCTTCGGGCCTTCCATCAAG |  |
| DatsR-F2 | GATGGAAGGCCCGAAGATGGAGTCGCATTGATCC |  |
| DatsR*-*R2 | CGCGGATCC CAGGTCGACTCTAGAGGATCCATCGGT  GCTGAACTTTGCGA (*Bam*HI) |  |
| Dncgl0884-F1 | GGAAGATCTTCAACTCATCAAGGTCGGTGCGGG (*Bgl*II) | To generate pK18*mobsacB-*Δ*ncgl0884* |
| Dncgl0884-R1 | CTCGCCCCAATTGGGCAGCAAATG |  |
| Dncgl0884-F2 | CATTTGCTGCCCAATTGGGGCGAGATGGCTACGCACTCACCGTTGAGG |  |
| Dncgl0884*-*R2 | CCCAAGCTTTCGCCAAACTTTTGATAGCGTAGC (*Hin*dIII) |  |
| Dncgl0887-F1 | CGCGGATCCTGTCTTGGAAGCATTTCACGGTGG (*Bam*HI) | To generate pK18*mobsacB-*Δ*ncgl0887* |
| Dncgl0887-R1 | AGGGTCTTGCCTTCCGGTGCCTG |  |
| Dncgl0887-F2 | CAGGCACCGGAAGGCAAGACCCTTTTCAAGCACGGTGCCCGCGTGG |  |
| Dncgl0887*-*R2 | CCCAAGCTTATGAACTCAAGCAACGGAGCAATG (*Hin*dIII) |  |
| *ncgl0884* _com_-F | CGCGGATCCAAAGGAGGACAACCATGGAAATTCCGCTGCCCACATC (*Bam*HI) | For cloning *ncgl0884* into pXMJ19 |
| *ncgl0884* _com_-R | CCCAAGCTTCTAGCCCACCTTTGTTAAATGCTC (*Eco*RI) |  |
| *ncgl0887* _com_-F | CCGGAATTCAAAGGAGGACAACCGTGGCGAAATTCCTGTATAAGT (*Eco*RI) | For cloning *ncgl0887* into pXMJ19 |
| *ncgl0887* _com_-R | CCCAAGCTTCTACATTTTCCTTCAGTTCCTC (*Hin*dIII) |  |
| Oncgl0887-F | CGGAATTCGTGGCGAAATTCCTGTATAAGTTAGGC (*Eco*RI) | To generate pET28a-*ncgl0887* |
| Oncgl0887-R | CCCAAGCTTCTAAGCCCCGACACCTACATTTTCC (*Hin*dIII) |  |
| OatsR-C123S-F | CTTGACCCCATCAAG*A*GTGTTATCGATAAGG | To generate *atsR:C123S* DNA fragment |
| OatsR-C123S-R | CCTTATCGATAACAC*T*CTTGATGGGGTCAAG |  |
| OatsR-C187S-F | TGATCCGTCGGGCA*A*GCGCCTGGATTAAG | To generate *atsR:C187S* DNA fragment |
| OatsR-C187S-R | CTTAATCCAGGCGC*T*TGCCCGACGGATCA |  |
| pET28aMCS-F | CCGGCGTAGAGGATCGAGATCTCG | To generate *atsR:C187S* DNA fragment |
| pET28aMCS-R | AGTGTTGTTCCAGTTTGGAACAAG |  |
| *P* *_ncgl0887_*-F | TCCCCCGGGTTACTGCTTAATTGGCGCCTCGGTG (*Sma*I) | To generate pK18*mobsacB-P _ncgl0887_::lacZY* |
| *P _ncgl0887_*-R | ACTAGTCAGGAATTTCGCCACGATTATC (*Spe*I) |  |
| lacZY-F1 | GATAATCGTGGCGAAATTCCTGACTAGT ATGACCATGATTACGGATTC(*Spe*I) |  |
| lacZY-R | AAAACTGCAGCAGACAACACCGAACCGAATCC (*Pst*I) |  |
| *P_ncgl0884_*-F | TCCCCCGGGCATGAGGAATGTTTCCCACTGTTGC (*Sma*I) | To generate pK18*mobsacB-P_ncgl0884_::lacZY* |
| *P_ncgl0884_*-R | CAGCGGAATTTCCATACCTTTAAG |  |
| lacZY-F2 | CTTAAAGGTATGGAAATTCCGCTGACTAGT ATGACCATGATTACGGATTC(*Spe*I) |  |
| Qncgl0884-F | GCCGCTCGACGATGATCTGATCAC | RT-PCR |
| Qncgl0884-R | GGTCAGTTCCAGGCTTCAGCGC |  |
| *P_ncgl0887_*-mutation-F | GATTTGAACGCGAACAACACGTTTTAACG | To produce 768-bp *P_ncgl0887M_* |
| *P_ncgl0887_*-mutation-R | CGTTAAAACGTGTTGTTCGCGTTCAAATG |  |
| QatsR-F | GTGTCGGGGCTTAGAGAAAC | RT-PCR |
| QatsR-R | GGGCCTTCCATCAAGGCGATC |  |
| Qncgl0887-F | CCGTTGTGGATCCTGTGTTGGC | RT-PCR |
| Qncgl0887-R | GTGACCTTCTCACGATCCTCTG |  |
| Encgl0887-F | GCCCACAGCATAGAACTAAAACAG | To produce the 232-bp EMSA promoter DNA |
| Encgl0887-R | GTTGACTGTTCTTCTGGGCTTTCC |  |
| Control-F1 | CAGGGTGTGGCTGCTCAGATG | To produce the 232-bp EMSA control DNA |
| Control-R1 | GTTGTAGACAACAGTCAGATC |  |
| Encgl0884-F | CGAATCCAATAGAACTAGCAACCG | To produce the 205-bp EMSA promoter DNA |
| Encgl0884-R | GTGGGCAGCGGAATTTCCATACC |  |
| Control-F2 | GAGTGGGTCAGCACGCGCGGTG | To produce the 205-bp EMSA control DNA |
| Control-R2 | CTTCGATGAGCGTGCGACGGATAC |  |
| 16 S rRNA-F | ACCCTTGTCTTATGTTGCCAG | RT-PCR |
| 16 S rRNA-R | TGTACCGACCATTGTAGCATG |  |

Underlined sites indicated restriction enzyme cutting sites added for cloning. Letters in italic denoted the mutation sites in overlap PCR for site-directed mutation.


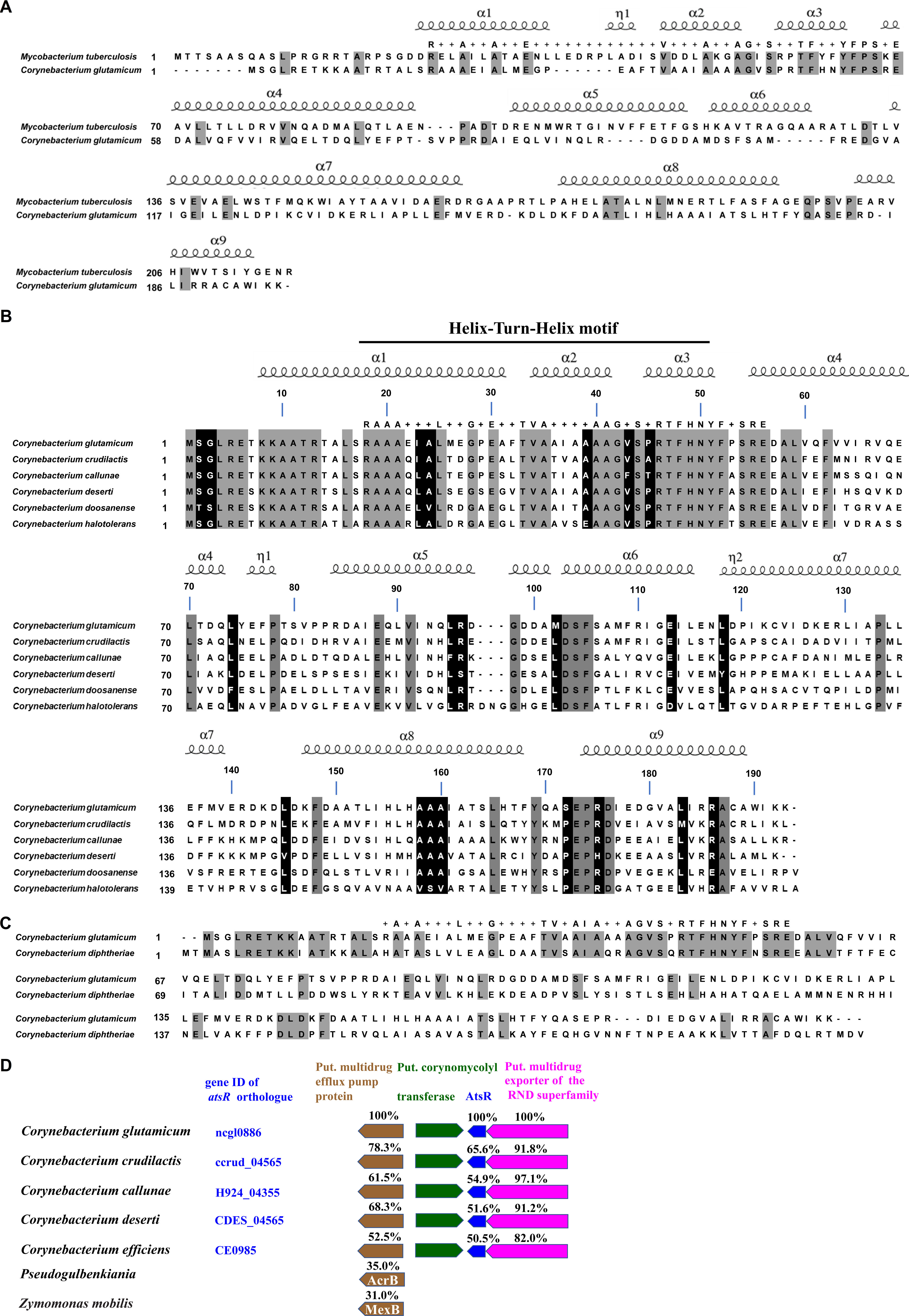


**Figure** **S1** **Multiple sequence alignment.** (**A**) Sequence alignment of AtsR with the TetR-type protein EthR from *Mycobacterium tuberculosiss* and secondary structure assignment*.* Amino acids identical were shaded in *gray* background. Reference sequences were retrieved from the NCBI Database, including *C. glutamicum* ATCC AtsR (CAF19629) and *M. tuberculosis* EthR (AJF05222). The amino acids of the N-terminal region of the EthR protein (amino acids 29-73) identical to those of the N-terminal region in the AtsR protein (amino acids 18-57) were shown above the alignment. (**B**) Sequence alignment of AtsR with TetR-type protein from several species of the genera *Corynebacterium* and secondary structure assignment of AtsR protein*.* Amino acids identical in all sequences were shaded in *gray* background, other conserved amino acids in black background. Reference sequences were retrieved from the NCBI Database, including *C. glutamicum* ATCC AtsR (CAF19629), *C. crudilactis* TetR (A ANE03558); *C. callunae* TetR (AGG66317); *C. deserti* TetR (ALC05358); *C. doosanense* TetR (AIT59905); *C. halotolerans* TetR (AGF71927). The N-terminal region of the AtsR proteins (amino acids 18-57) showed clear similarity to the N-terminal part of the TetR family of the other regulatory proteins (TetR-N; PFAM00440). The amino acids identical to those of the N-terminal region in the AtsR protein were shown above the alignment. (**C**) Multiple sequence alignment of AtsR with TetR-type protein from *C. diphtheriae*. Residues that were identical in 2 sequences were depicted on the gray background. Reference sequences were retrieved from the NCBI Database, including *C. glutamicum* ATCC AtsR (CAF19629); *C. diphtheriae* TetR (AEX46079). The amino acids of the N-terminal region of the *C. diphtheriae* TetR protein (amino acids 20-59) identical those of the N-terminal region in the AtsR protein were shown above the alignment. (**D**) Genomic organization of the *atsR* gene in several *corynebacterial* species. Homologous genes were presented in the same color. The amino acid sequence similarities (expressed as percentages) of the corresponding proteins in *C. glutamicum* with those in other strains were shown. Reference sequences were retrieved from the NCBI Database, including *Pseudogulbenkiania* sp. NH8B (BAK77170) and *Zymomonas mobilis* subsp. mobilis CP4 (AHJ72432).


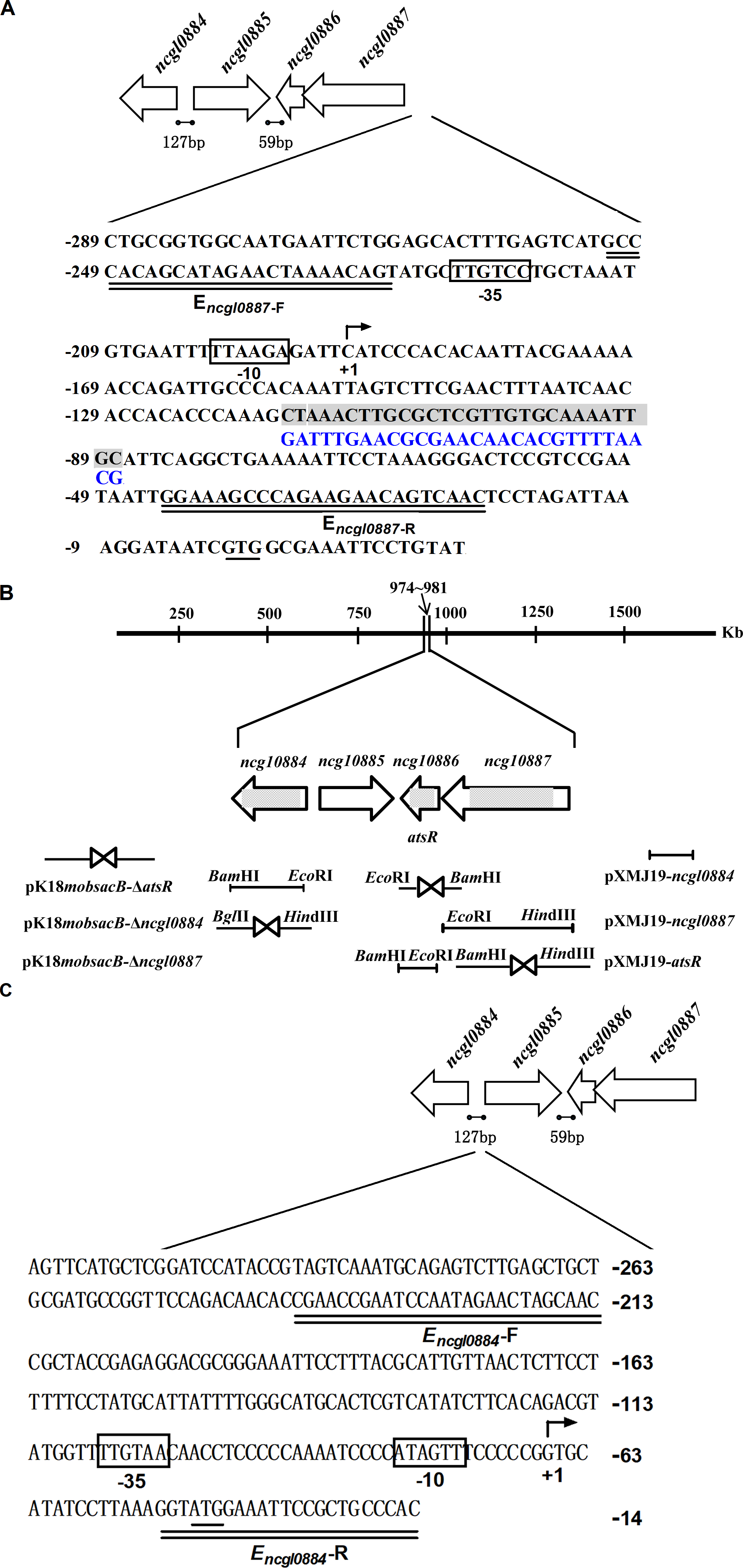


**Figure S2** **Detailed genetic maps.** (**A)** The promoter region was upstream of the *ncgl0887*-*atsR* operon. Gray shaded box indicated the 29 bp sequences, which was assumed to contain the AtsR binding site. The deduced -35 and -10 promoter regions found by PROM-Prediction of bacterial promoters were boxed. The start codon of *ncgl0887* was underlined. The sequences used to replace the 29 bp sequences were shown in blue below the promoter sequence. EMSA DNA promoter fragment was obtained using primers E_ncgl0887_-F and E_ncgl0887_-R, which was double underlined. (**B)** Physical map of *ncgl0884* and *ncgl0887*-*atsR* operon in *Corynebacterium glutamicum* RES167 parental strain and construction of plasmids for gene disruption (pK18*mobsacB* derivatives) or complementation (pXMJ19 derivatives). Open reading frames (ORFs) were marked by open arrows, and the deleted regions were in grey. The restriction sites were indicated. **(C)** The promoter region was upstream of *ncgl0884* gene. The deduced -35 and -10 promoter regions found by PROM-Prediction of bacterial promoters were boxed. The start codon of *ncgl0884* was underlined. EMSA DNA promoter fragment was obtained using primers Encgl0884-F and Encgl0884-R, which was double underlined.


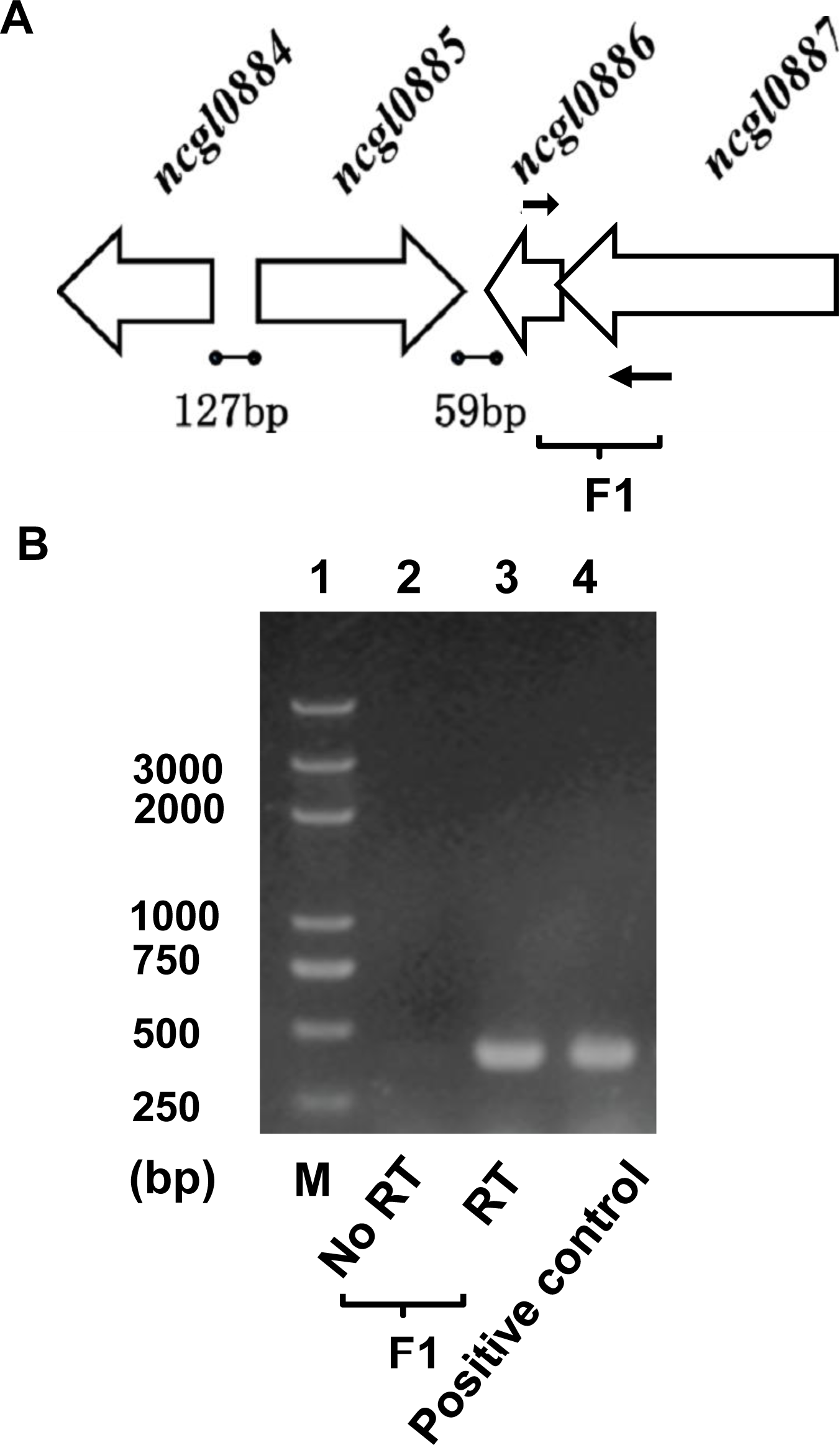


**Figure S3 Assays for the *ncgl0887*-*atsR* co-transcription by reverse transcription PCR. (A)** The operon structure of *ncgl0887*-*atsR* primer where designed for assays and indicated by blank arrows. **(B)** Reverse transcription PCR assays for *ncgl0887*-*atsR* co-transcription. Lane 1, DNA marker; Lane 2, negative control PCR reactions omitted the initial reverse transcription step (No-RT). lane 4, positive control using genomic DNA as template. PCR procedure was as follows: reactions were denatured 95 °C for 50 s, annealed at 58 °C for 40 s, extended at 72 °C for 30 s, and repeated 30 cycles.


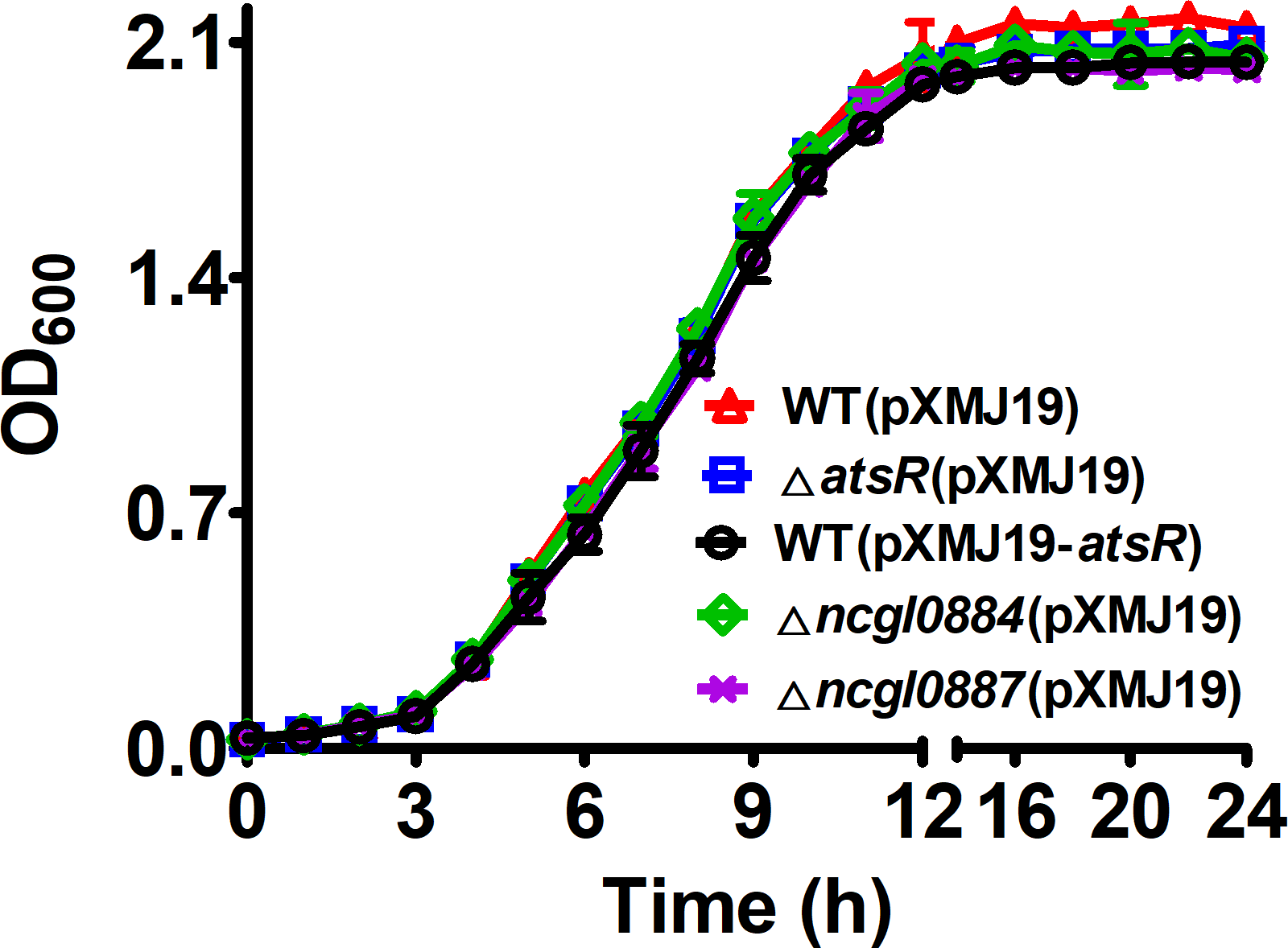


**Figure S4** Growth curves of the WT(pXMJ19) strain (the *C. glutamicum* RES167 parental strain transformed with the empty plasmid pXMJ19), the Δ*atsR*(pXMJ19) mutant (the *atsR* deletion mutant expressing pXMJ19), the WT(pXMJ19-*atsR*) strain (*C. glutamicum* RES167 parental strain expressing the wide-type (WT) *atsR* gene in the shuttle vector pXMJ19), Δ*ncgl0884*(pXMJ19) mutant (the *ncgl0884* deletion mutant expressing pXMJ19), and Δ*ncgl0887*(pXMJ19) mutant (the *ncgl0884* deletion mutant expressing pXMJ19) under normal conditions. The growth of the indicated strains in LB media was monitored by measuring OD_600_ at indicated time points.


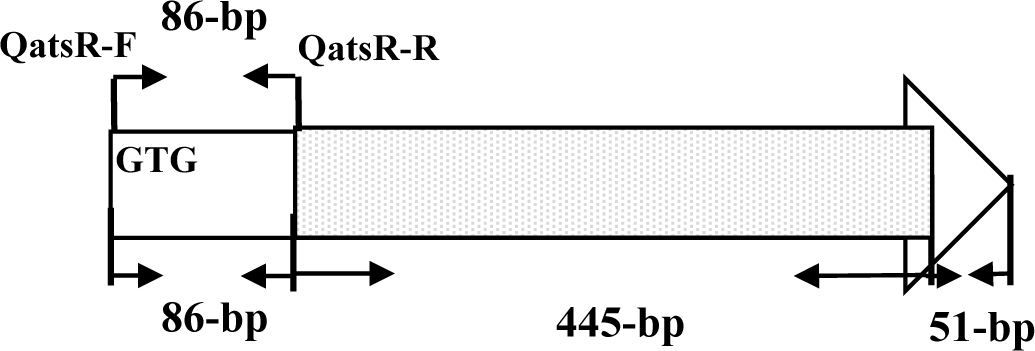


**Figure S5 86-bp *atsR* transcript (from the translational start codon (GTG) of *atsR* gene to 86th nucleotide) was amplified from the remaining *atsR* ORF (open reading frame) in Δ*atsR* mutant with primers QatsR-F and QatsR-R.** *atsR* ORF was marked by open arrows and the deleted region was in gray.


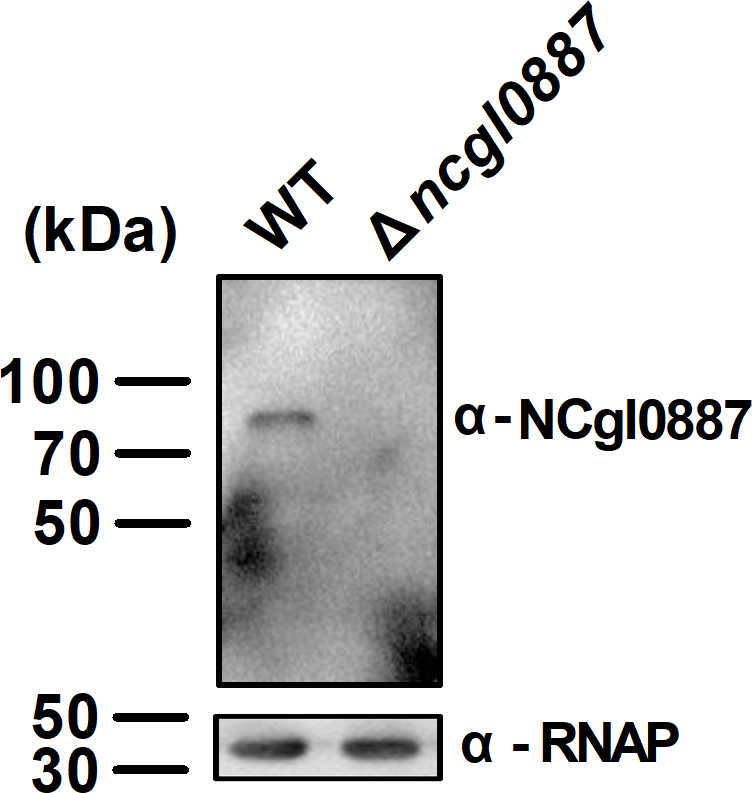


**Figure** **S6 The NCgl0887 was examined in *C. glutamicum*.** Anti-NCgl0887 antibody detected a single protein with a mobility consistent with the predicted size that was absent from Δ*ncgl0887* mutants. Antibody to RNAP was used as a loading control.


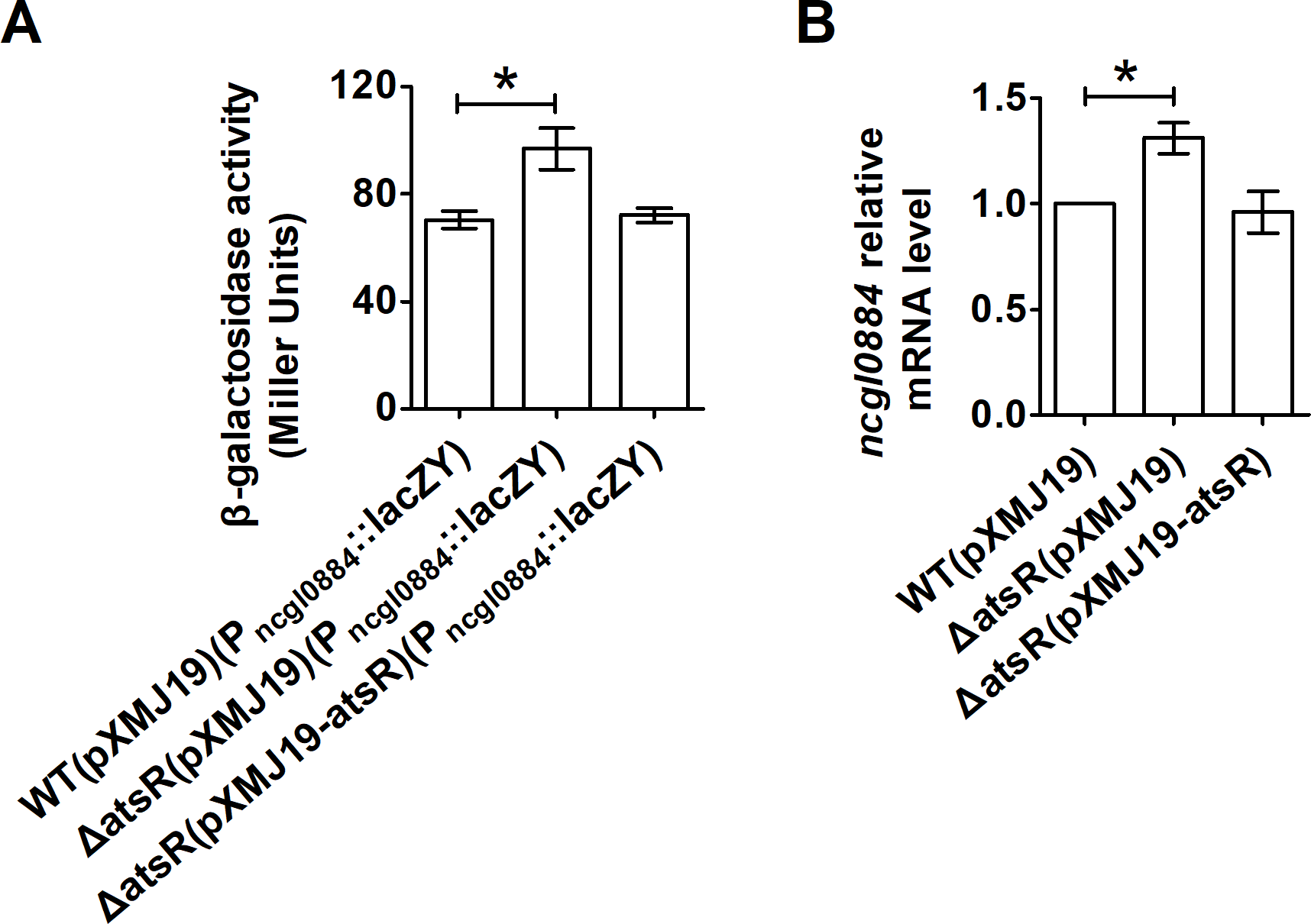


**Figure** **S7 Negative regulation of *ncgl0884* expression by AtsR.** (**A)** β-galactosidase activity analysis of the *ncgl0884* promoter was performed using the transcriptional *P_ncgl0884_::lacZY* chromosomal fusion reporter expressed in WT(pXMJ19), Δ*atsR*(pXMJ19) mutant, and complementary Δ*atsR*(pXMJ19-*atsR*) strains. **(B)** qRT-PCR assay was performed to analyze the *ncgl0884* expression in WT(pXMJ19), Δ*atsR*(pXMJ19), and complementary Δ*atsR*(pXMJ19-*atsR*) strains. The mRNA levels were presented relative to the value obtained from WT(pXMJ19) strains. Relative transcript levels of WT(pXMJ19) strains were set at a value of 1.0. Data show the averages of three independent experiments, and error bars indicated the SDs from three independent experiments. *: P≤0.05.


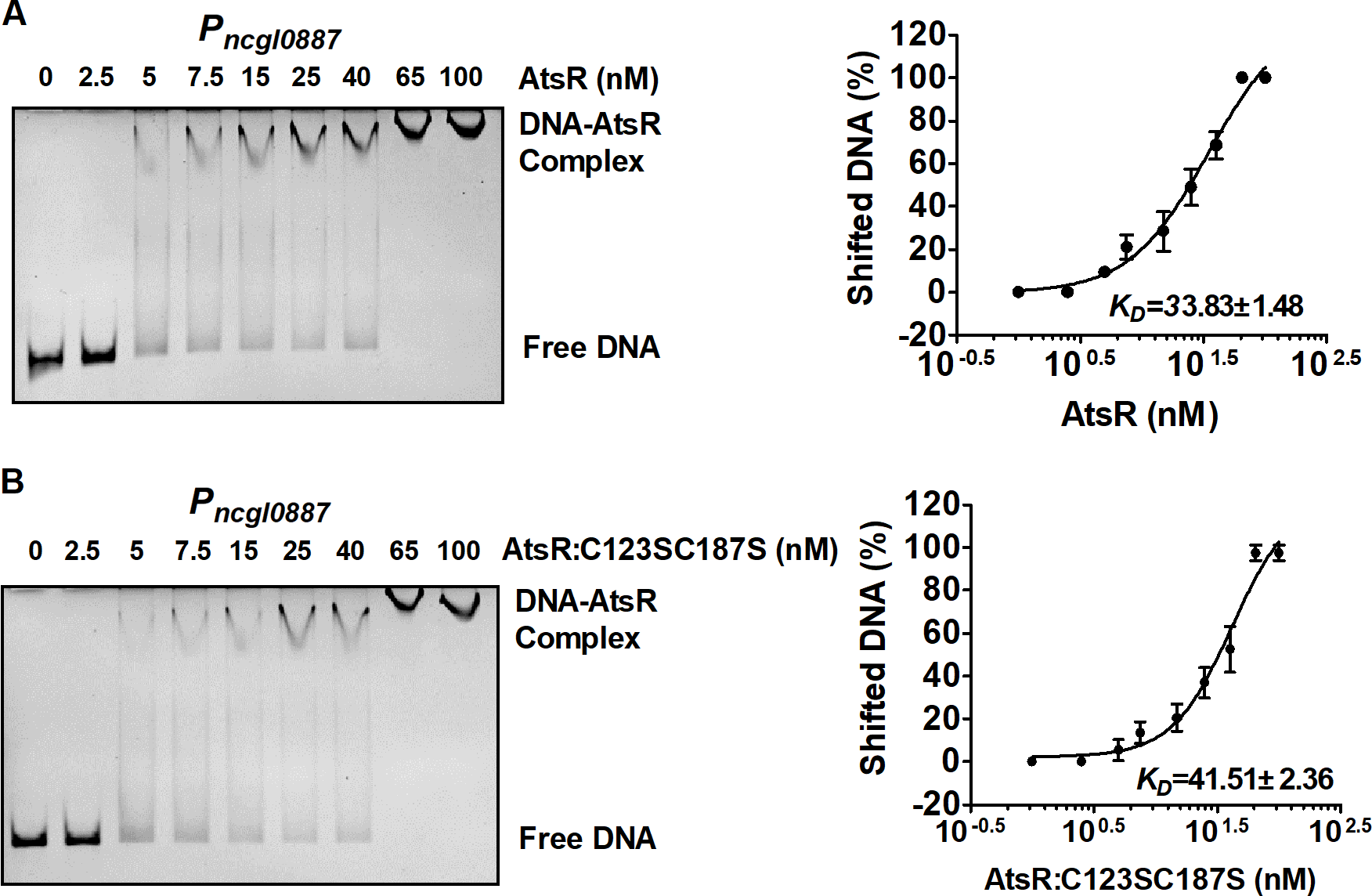


**Figure** **S8** **Determination of the apparent *K_D_* values of AtsR and AtsR:C123SC187S for *P_ncgl0887_*.** *P_ncgl0887_* was incubated with increasing AtsR (A) or AtsR:C123SC187S (B) concentrations, resolved on a 8% native PAGE, and stained with GelRed^TM^. At least three independent gels were performed for each binding site. The bands were quantified using ImageQuant software (GE Healthcare), and the percentage of shifted DNA was calculated from three independent gels. These values were plotted against the AtsR or AtsR:C123SC187S concentration in log_10_ scale, and a sigmoidal fit was performed. The turning point of the curve was defined as the apparent *K_D_* value.


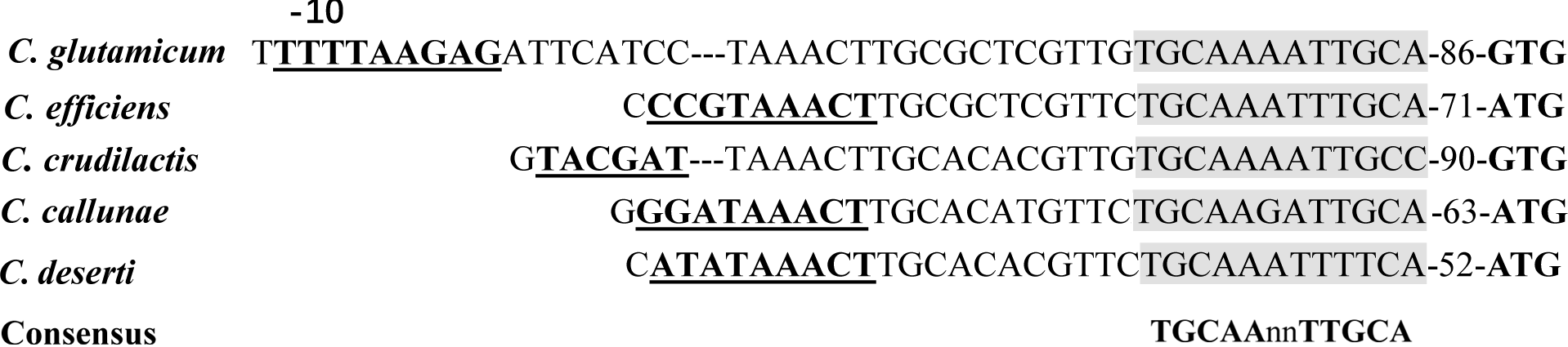


**Figure** **S9 Sequence of the promoter region of *C. glutamicum ncgl0887-atsR* operon aligned to putative promoter regions from other *Corynebacterium* species.** Indicated were the start of the *ncgl0887* coding region (start codons black bold), putative -10 (underlined and black bold), and the AtsR binding site (shaded in gray) for the *C. glutamicum* *ncgl0887-atsR* operon. As shown by the alignment, also the other species possessed putative AtsR binding sites in the *ncgl0887* upstream region. The binding site represented a perfect inverted repeat in five *Corynebacterium* species with the consensus sequence 5΄-TGCAA-N_2_-TTGCA-3΄.


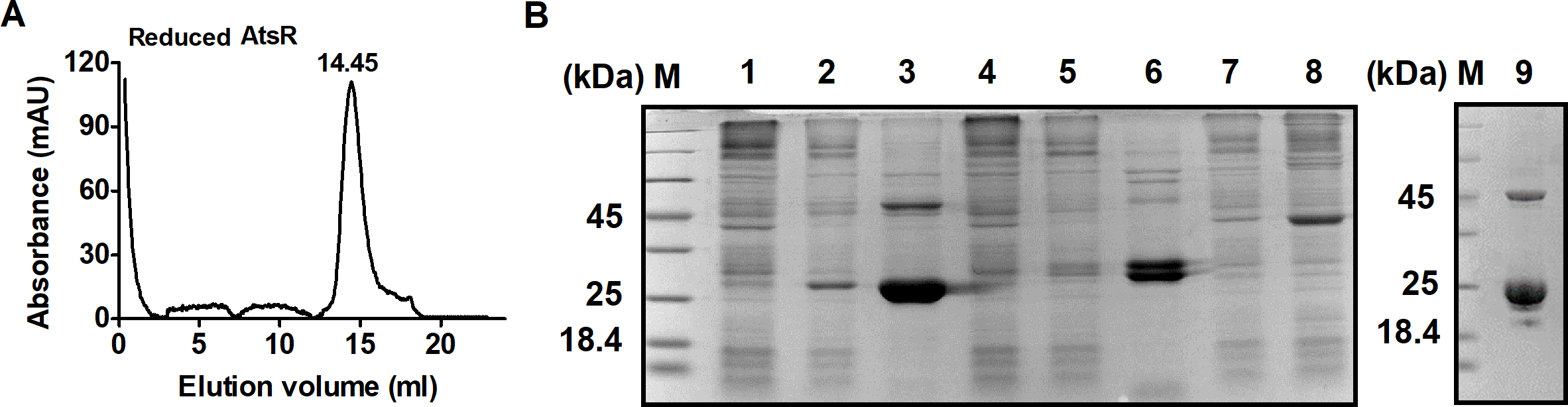


**Figure S10 Purification of AtsR.** (A) Elution of reduced AtsR (20 μM AtsR was incubated with 50 mM DTT) from size exclusion column. (B) Coomassie-stained SDS-PAGE of His_6_-AtsR, His_6_-PUP1, His_6_-SUMO-AtsR, and AtsR. M, broad-range protein marker; lane 1, crude extract of BL21(pET28a*-atsR*) strain without IPTG induction; lane 2, crude extract of BL21(pET28a-*atsR*) strain with induction; lane 3, purified His_6_-AtsR protein; lane 4, crude extract of BL21(pET28a*-pup1*) strain without IPTG induction; lane 5, crude extract of BL21(pET28a-*pup1*) strain with induction; lane 6, purified His_6_-PUP1 protein; lane 7, crude extract of BL21(pET28a*-*SUMO*-atsR*) strain without IPTG induction; lane 8, crude extract of BL21(pET28a-pET28a*-*SUMO*-atsR*) strain with induction; lane 9, purified AtsR protein.
